# Novel and improved *Caenorhabditis briggsae* gene models generated by community curation

**DOI:** 10.1186/s12864-023-09582-0

**Published:** 2023-08-25

**Authors:** Nicolas D. Moya, Lewis Stevens, Isabella R. Miller, Chloe E. Sokol, Joseph L. Galindo, Alexandra D. Bardas, Edward S. H. Koh, Justine Rozenich, Cassia Yeo, Maryanne Xu, Erik C. Andersen

**Affiliations:** 1 Department of Molecular Biosciences, Northwestern University, 4619 Silverman Hall 2205 Tech Drive, Evanston, IL 60208 USA; 2 Interdisciplinary Biological Sciences Program, Northwestern University, Evanston, IL 60208 USA; 3 Tree of Life, Wellcome Sanger Institute, Cambridge, UK

**Keywords:** Curation, Gene models, *C. briggsae*, Apollo, Community curation

## Abstract

**Background:**

The nematode *Caenorhabditis briggsae* has been used as a model in comparative genomics studies with *Caenorhabditis elegans* because of their striking morphological and behavioral similarities. However, the potential of *C. briggsae* for comparative studies is limited by the quality of its genome resources. The genome resources for the *C. briggsae* laboratory strain AF16 have not been developed to the same extent as *C. elegans*. The recent publication of a new chromosome-level reference genome for QX1410, a *C. briggsae* wild strain closely related to AF16, has provided the first step to bridge the gap between *C. elegans* and *C. briggsae* genome resources. Currently, the QX1410 gene models consist of software-derived gene predictions that contain numerous errors in their structure and coding sequences. In this study, a team of researchers manually inspected over 21,000 gene models and underlying transcriptomic data to repair software-derived errors.

**Results:**

We designed a detailed workflow to train a team of nine students to manually curate gene models using RNA read alignments. We manually inspected the gene models, proposed corrections to the coding sequences of over 8,000 genes, and modeled thousands of putative isoforms and untranslated regions. We exploited the conservation of protein sequence length between *C. briggsae* and *C. elegans* to quantify the improvement in protein-coding gene model quality and showed that manual curation led to substantial improvements in the protein sequence length accuracy of QX1410 genes. Additionally, collinear alignment analysis between the QX1410 and AF16 genomes revealed over 1,800 genes affected by spurious duplications and inversions in the AF16 genome that are now resolved in the QX1410 genome.

**Conclusions:**

Community-based, manual curation using transcriptome data is an effective approach to improve the quality of software-derived protein-coding genes. The detailed protocols provided in this work can be useful for future large-scale manual curation projects in other species. Our manual curation efforts have brought the QX1410 gene models to a comparable level of quality as the extensively curated AF16 gene models. The improved genome resources for *C. briggsae* provide reliable tools for the study of *Caenorhabditis* biology and other related nematodes.

**Supplementary Information:**

The online version contains supplementary material available at 10.1186/s12864-023-09582-0.

## Background

The undisputed popularity of the free-living nematode *Caenorhabditis elegans* as a highly tractable model organism is facilitated by the vast collection of genetic and genomic resources that have been arduously maintained and improved by the research community. *C. elegans* possesses one of the highest quality metazoan genome assemblies with extensively validated models of its protein-coding genes. Similar efforts to generate and improve genomic resources for other species in the *Caenorhabditis* genus have enabled comparative studies that extended our understanding of genetics, development, and evolution [1–5]. The nematode *Caenorhabditis briggsae* has been a major focus of such comparative studies because of its striking morphological and behavioral similarities to *C. elegans*. Both species reproduce primarily by self-fertilization, have nearly identical body plans, are globally distributed, and share similar ecology [6–8]. Conversely, genomic studies have shown that *C. briggsae* populations are stratified into distinct phylogeographic groups and maintain higher genetic diversity than *C. elegans* [9]. Despite the usefulness of *C. briggsae* to study *Caenorhabditis* biology, its genomic resources have not been developed to the same extent as *C. elegans*. The draft genome assembly of the *C. briggsae* laboratory strain, AF16, was first assembled in 2003 using a combination of whole-genome shotgun sequencing and a physical map based on fosmid and bacterial artificial chromosome libraries [10]. In 2014, an updated version of the AF16 genome was made available (named ‘cb4’), which resolved many assembly artifacts by using a high-resolution recombination map generated from recombinant inbred lines [11]. To date, the AF16 ‘cb4’ genome is available in public databases and widely used in genomic studies but remains highly fragmented, with thousands of unresolved gaps and hundreds of misoriented, mis-scaffolded, or unplaced sequences [5, 12]. Moreover, unlike the gene models for the *C. elegans* laboratory strain N2, the AF16 gene models have not been extensively investigated and likely possess numerous structural and coding sequence errors. Although efforts to identify and correct errors in the AF16 gene models have led to substantial improvements in accuracy and completeness, many loci remain inaccurate or cannot be corrected because of inconsistencies in the genome assembly [13].

Recently, advancements in chromosome conformation capture techniques, such as Hi-C, and novel long-read sequencing technologies offered by Oxford Nanopore Technologies (ONT) and Pacific Biosciences (PacBio) have enabled the rapid assembly of highly contiguous genomes. These technologies have been employed to repair the gaps in the `cb4` genome (‘cb4_improve’), and anchor unplaced scaffolds using an independent *de novo* assembly (‘cb4_SLR_HiC’) generated from PacBio SLR reads scaffolded with Hi-C data [12]. Despite these efforts, the improved AF16 genome still possesses over 3,500 gaps and over 200 unplaced scaffolds, and has yet to be made publicly available [12, 14]. Moreover, the errors in the AF16 gene models stemming from fragmented contigs and misplaced sequences are likely to persist in the improved AF16 genome.

In a previous study, we introduced a new high-quality genome assembly for the *C. briggsae* strain QX1410, a new reference strain isolated in the wild and closely related to AF16 [5]. The QX1410 genome assembly features chromosomally resolved contigs, defined chromosomal domains, and few unresolved gaps. Additionally, we generated preliminary protein-coding gene models using gene prediction tools that leverage short- and long-read RNA sequencing data. Here, we focused on the identification and repair of errors caused by automated gene predictions in the new *C. briggsae* reference strain, QX1410. To accomplish this large manual curation task, we assembled a team of nine high school, undergraduate, and graduate students. This team was trained to interpret transcriptomic data and interact with the Apollo genomic annotation editor [15], which enables easy viewing of primary transcriptomic data, along with an effective toolkit to interpret and curate gene models. We designed an extensive protocol with step-by-step workflows to identify and repair the most common errors found in automated gene predictions using RNA alignments. In the span of one year, we manually curated and revised over 22,000 loci to provide improved protein-coding gene models for the new *C. briggsae* reference genome. We exploited the high-quality gene models of the *C. elegans* laboratory strain N2 and the high level of protein sequence conservation between *C. elegans* and *C. briggsae* to assess the quality of the protein-gene models in the QX1410 and AF16 strains.

## Results

### Short- and long-read RNA alignments can be used to correct structural errors in protein-coding gene predictions

**Fig. 1:**
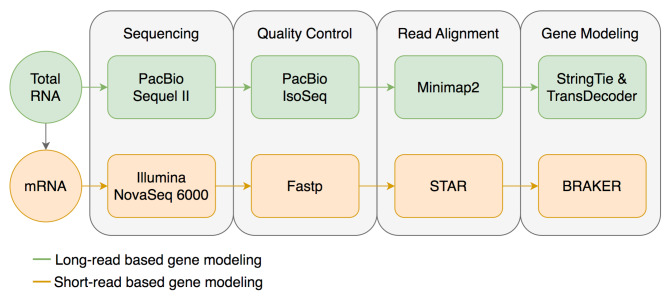
Data processing schematic. A flowchart describing the data processing steps required prior to the manual curation process. RNA is first sequenced using both PacBio and Illumina platforms. PacBio long reads are trimmed and refined using the IsoSeq pipeline, aligned to the reference genome using Minimap2, assembled into non-redundant transcripts using StringTie, and ORFs predicted using TransDecoder. Illumina short reads are trimmed using Fastp, aligned to the reference genome using STAR, and protein-coding genes are predicted using BRAKER

**Fig. 2:**
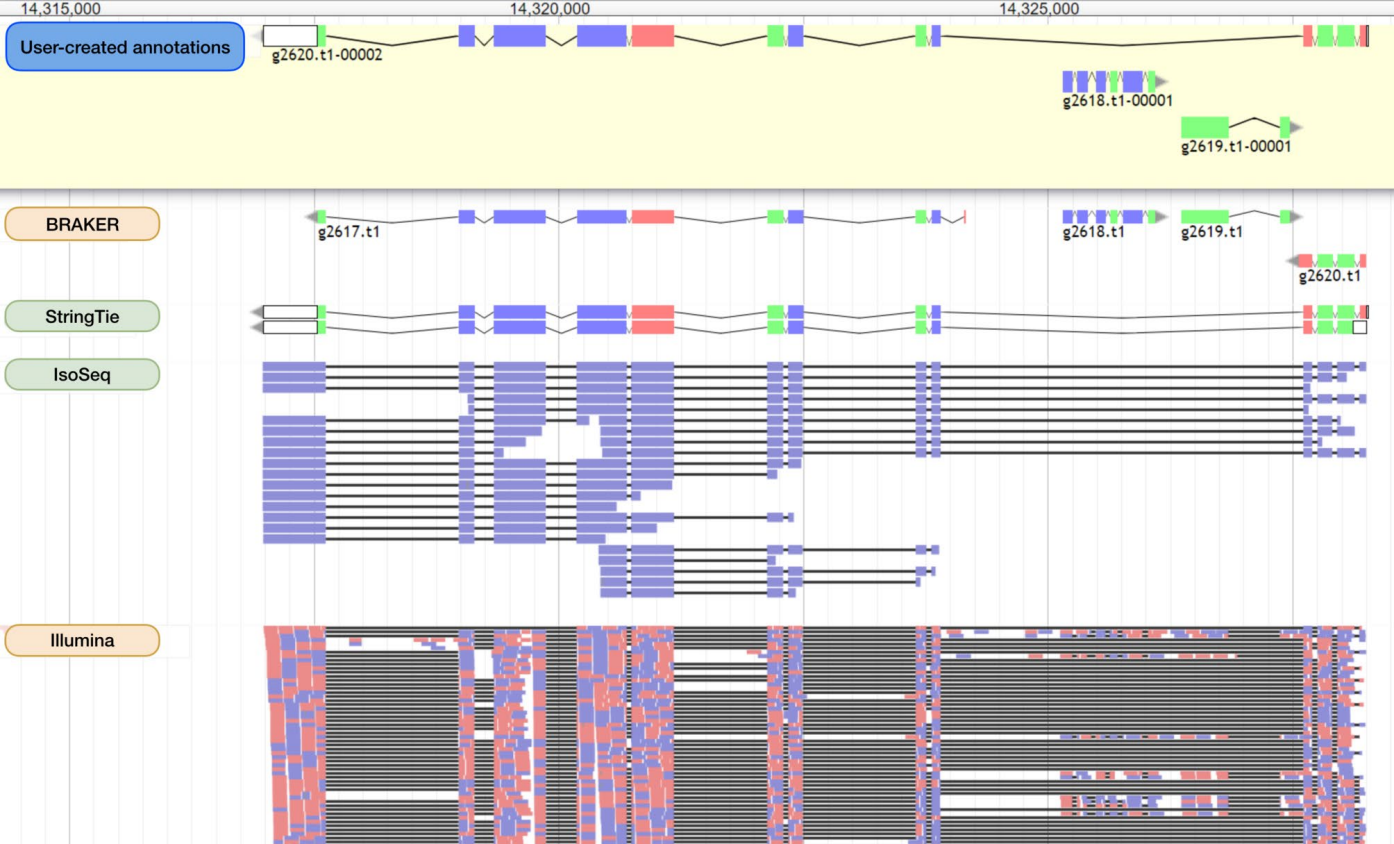
Identification of structural errors in protein-coding gene predictions. A screen capture of the Apollo genome annotation editor showing a set of manually curated genes (located on chromosome X from 14,314,000 to 14,325,000) and their underlying evidence. Four individual tracks are displayed from top to bottom: BRAKER gene models, StringTie gene models, PacBio Iso-Seq refined transcript alignments, and paired-end Illumina RNA-seq alignments. The final set of curated gene models is displayed in the top box shaded in yellow labeled ‘User-created Annotations’. Both Illumina and PacBio RNA data suggest that the two BRAKER genes at the ends of this region were incorrectly split. StringTie models resolve the incorrect split but lack the two internal genes on the opposite strand (g2618.t1 and g2619.t1), because they lack long-read RNA coverage. We kept the StringTie model that best matches the RNA evidence and added the two internal genes on the opposite strand predicted by BRAKER and supported by short-read RNA-seq. Curated and predicted gene models are colored by coding sequence phase. Illumina and IsoSeq alignments are colored by strand orientation

**Fig. 3:**
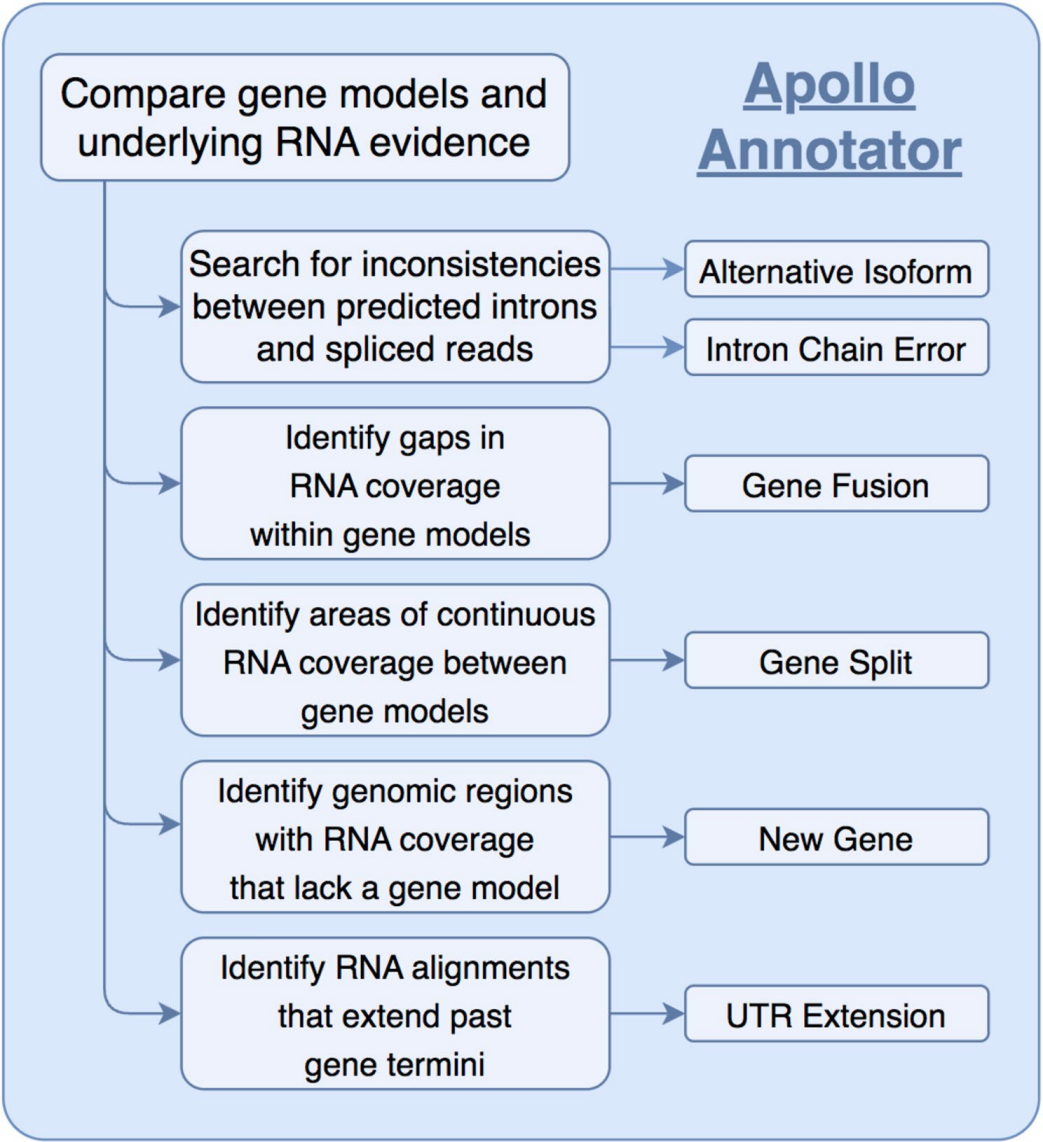
Schematic of manual curation process. Schematic describing the main logical steps to classify types of curation errors based on a comparison between RNA evidence and modeled genes from gene prediction tools. RNA evidence is sufficient to discern if a predicted gene model has major structural errors (e.g., gene fusion, gene split, intron chain error) or requires minor edits (e.g., additional isoform, UTR extension). Genomic regions with RNA coverage that lack a gene prediction were used to manually model genes *de novo*

We aimed to identify and correct structural errors in every predicted gene in the QX1410 reference gene models by leveraging short- and long-read RNA sequence (RNA-seq) alignments. We extracted and pooled RNA from mixed-stage, stage-specific, male-enriched, or starved cultures to maximize transcript representation across all stages of development and both sexes. We sequenced the QX1410 transcriptome using both PacBio Single-Molecule Real-Time (SMRT) and Illumina platforms and refined the PacBio long reads into 95,177 high-quality transcripts using the IsoSeq pipeline [16]. We generated a set of long-read based gene models by identifying common intron chains and predicting open reading frames (ORFs) in assembled PacBio high-quality transcripts using StringTie and TransDecoder [17, 18]. Additionally, we generated a set of gene models from short-read RNA-seq alignments using BRAKER (Fig. 1) [19]. A merger of these long- and short-read based gene models was previously published along with the chromosomal genome assembly of QX1410 [5]. The manual curation process relied on the identification of differences between computationally generated gene models and underlying RNA read alignments. We loaded short- and long-read alignments and gene models as individual evidence tracks into the Apollo genome annotation editor. We complemented the high-depth Illumina RNA-seq data with the structural information from full-length PacBio transcripts to provide high-confidence gene models for every locus with RNA coverage (Fig. 2). When appropriate, we edited the structure of gene predictions to match the intron chain and gene termini best supported by RNA evidence. We manually curated 22,189 genes, amounting to a total of 32,278 transcripts (1.45 transcripts/gene). The total gene count in QX1410 is unusually higher than both AF16 and N2 (Table 1). This difference in gene count can be explained by *de novo* gene predictions that were kept during manual curation. If a gene model did not possess any underlying RNA evidence, we made the conservative choice to preserve the model, as we could not discern if the gene was real or simply not captured in our sequencing effort. Comparisons of protein sequences between QX1410 gene models indicates we modified the coding sequences of 8,064 genes during the curation process. From these modified genes, 390 (4.8%) were classified as a gene split, 919 (11.4%) were classified as gene fusion, 4,751 (58.9%) were classified as intron chain error (missing or additional exon, or exon-intron junction modification), and 2,004 (24.9%) were not classified because of missing manual entries in our curatorial records. Additionally, we modeled putative alternative splice isoforms when new splice sites were identified in the RNA alignments. We identified variation in spliced reads mapped to existing gene models and added 10,089 putative isoforms in 5,398 genes. We also annotated 50,131 preliminary UTRs, including 5’ UTRs for 58.9% of genes and 3’ UTRs for 64.8% of genes (Fig. 3). Examples of each type of curation, including workflows to correct different gene model error types, are provided in the curation protocol (Additional file 1).

### Protein-length accuracy analysis reveals improvements after manual curation

**Fig. 4:**
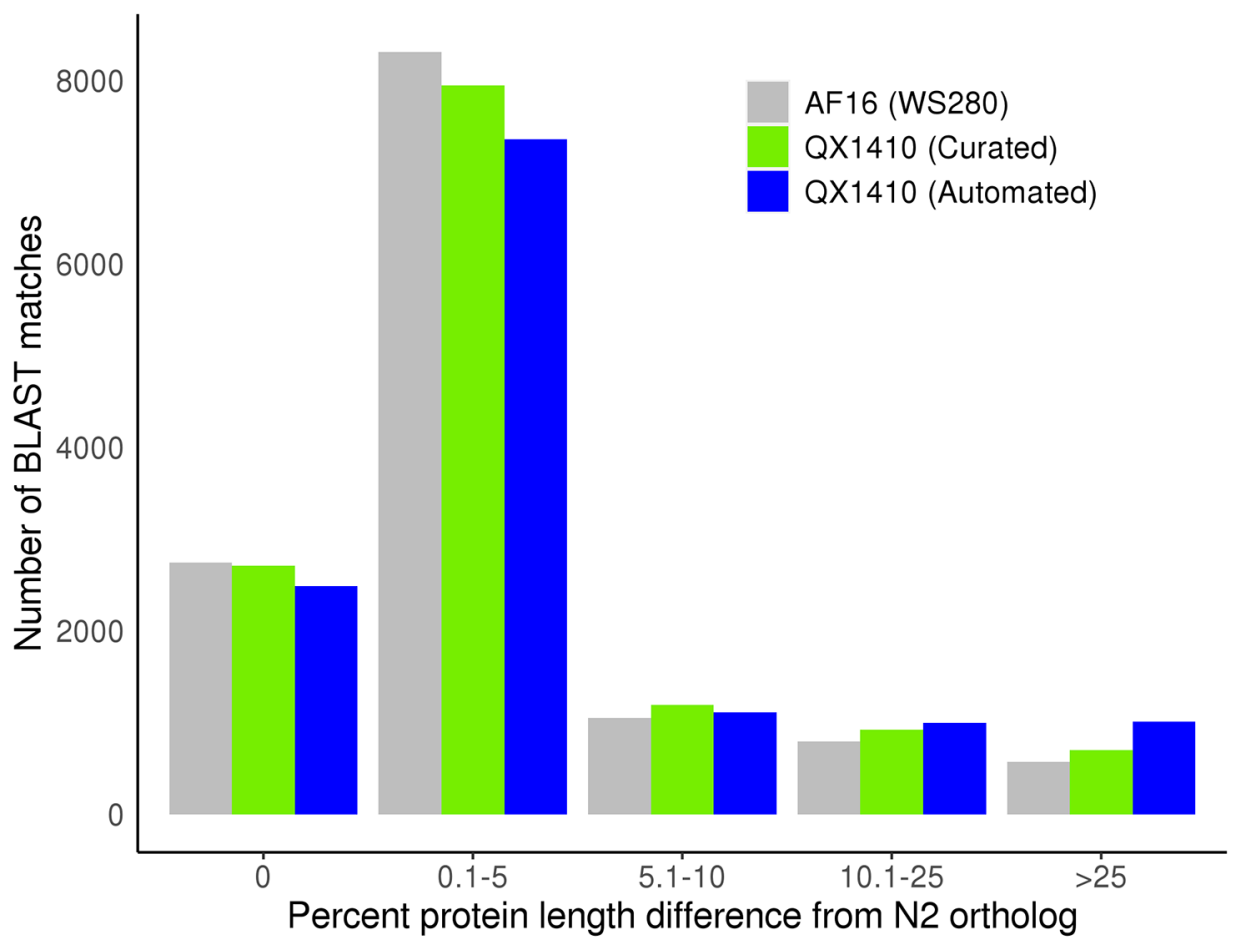
Protein-length accuracy of the ***C. briggsae*** gene models relative to reciprocal BLAST matches in ***C. elegans*** N2 protein sequences. The binned counts of protein-length accuracy values for both sets of QX1410 gene models (automated and curated) and the AF16 gene models are shown. We calculated the percent protein-length difference of each QX1410 gene model relative to their best reciprocal BLAST hit found in the N2 genome. We performed the same calculation for every gene in the AF16 (WS280) genome. A percent difference value of zero represents a *C. briggsae* gene with a protein length that perfectly matches its best reciprocal hit in the N2 genome. A percent difference value of 1 represents a *C. briggsae* gene with a protein length that is 1% shorter or longer than its best reciprocal hit in the N2 genome and is placed under the bin labeled ‘0.1-5’. The total number of shared genes compared in this BLAST analysis is 13,469

**Fig. 5:**
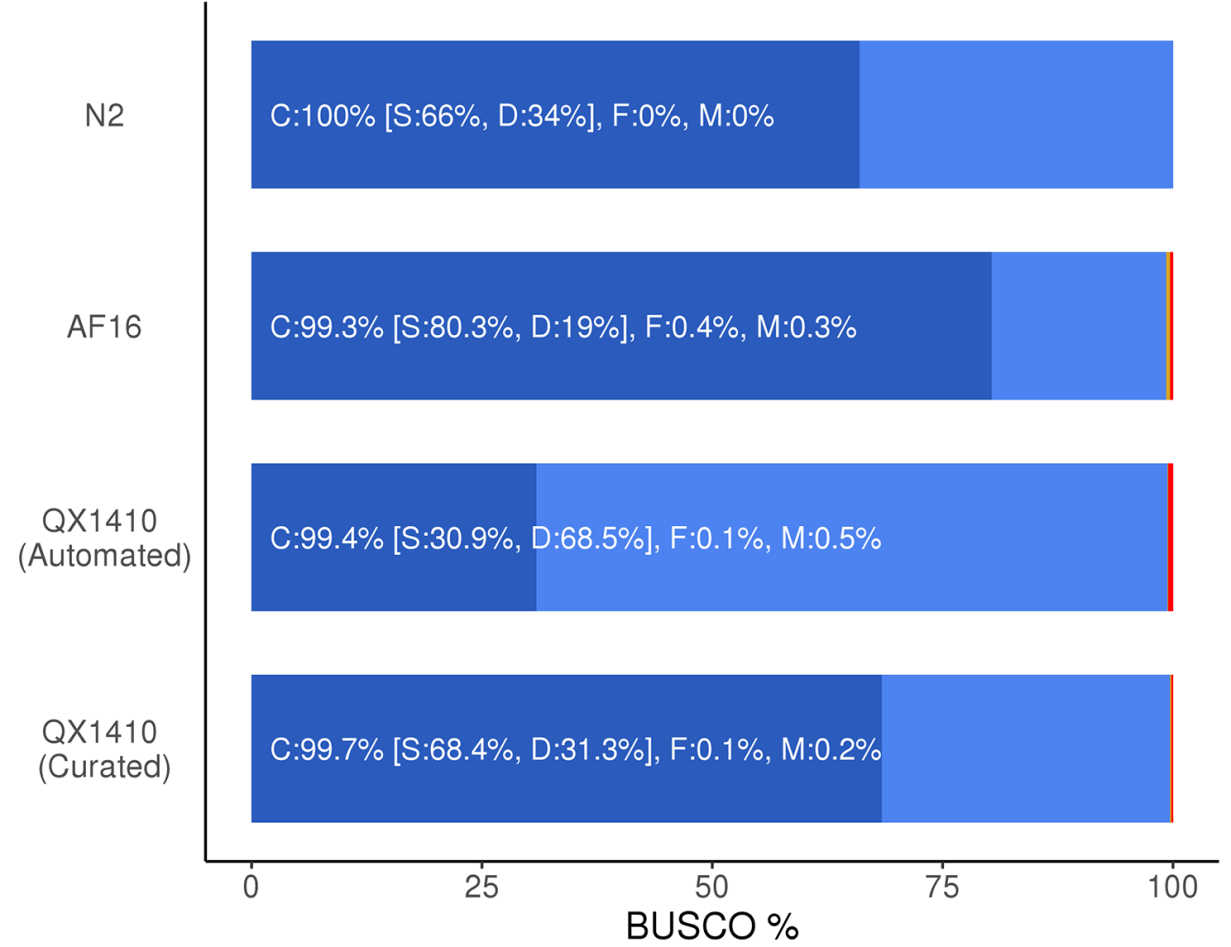
BUSCO assessment values for ***C. briggsae*** and ***C. elegans*** gene models. Stacked bar plot showing the percentage of complete, duplicated, fragmented, and missing BUSCOs for the *C. elegans* (N2) and *C. briggsae* (AF16, QX1410 automated, and QX1410 curated) proteomes. Breakpoints between each BUSCO classification are denoted by a change in color. Dark blue represents the complete BUSCOs, light blue represents the complete and duplicated BUSCOs, yellow represents the fragmented BUSCOs, and red represents the missing BUSCOs. The nematoda_odb10 database was used

To assess the quality of the manually curated *C. briggsae* gene models, we identified orthologous sequences between each *C. briggsae* gene set (QX1410 automated, QX1410 curated, and AF16) and the reference *C. elegans* strain N2 using a reciprocal best BLAST hit approach. The AF16 genome and proteome were retrieved from WormBase (WS280), which hosts the ‘cb4’ version of the AF16 genome resources. We identified the set of gene models in AF16 and QX1410 that share the same N2 ortholog, and compared the translated protein sequence length of each gene model against its corresponding N2 ortholog. Next, we estimated the ratio between a *C. briggsae* protein sequence and its best *C. elegans* reciprocal match, which we called ‘protein-length accuracy’. Finally, we counted the number of identical protein-length matches, matches that were within 5%, between 5% and 10%, between 10% and 25%, or over 25% of the protein length of its N2 ortholog. Our manual curation efforts led to an overall improvement in protein-length accuracy (Fig. 4). Specifically, we found a substantial increase in the number of identical, within 5%, and between 5% and 10% off their reciprocal BLAST matches (Fig. 4). Additionally, this improvement in protein-length accuracy was also accompanied by marginal improvements in BUSCO completeness. BUSCO completeness of the curated gene set was 99.7%, an improvement of 0.3% over the automated gene models, and 0.4% over AF16 (Fig. 5). The high BUSCO duplication values observed in both the curated and automated QX1410 gene models relative to AF16 gene models suggest an increase in alternative isoforms (Fig. 5). Many isoforms were modeled from variation in spliced reads from short-read RNA-seq, and certain splice variant combinations might not exist as correct gene isoforms. Considering that manual curation was highly effective at reducing BUSCO duplication in the QX1410 genome (from 68.5 to 31.5%), the availability of full-length transcripts assembled from long-read RNA sequencing played a major role at identifying incorrect or redundant isoforms (Fig. 5; Additional file 2, Fig. S1). Higher depth long-read RNA sequencing or other techniques specifically designed to maximize isoform discovery will be necessary to further identify and discard spurious isoforms that are still present in the current gene models.

### Protein-length analysis shows high concordance in protein-sequence length between the QX1410 and AF16 gene models

**Fig. 6:**
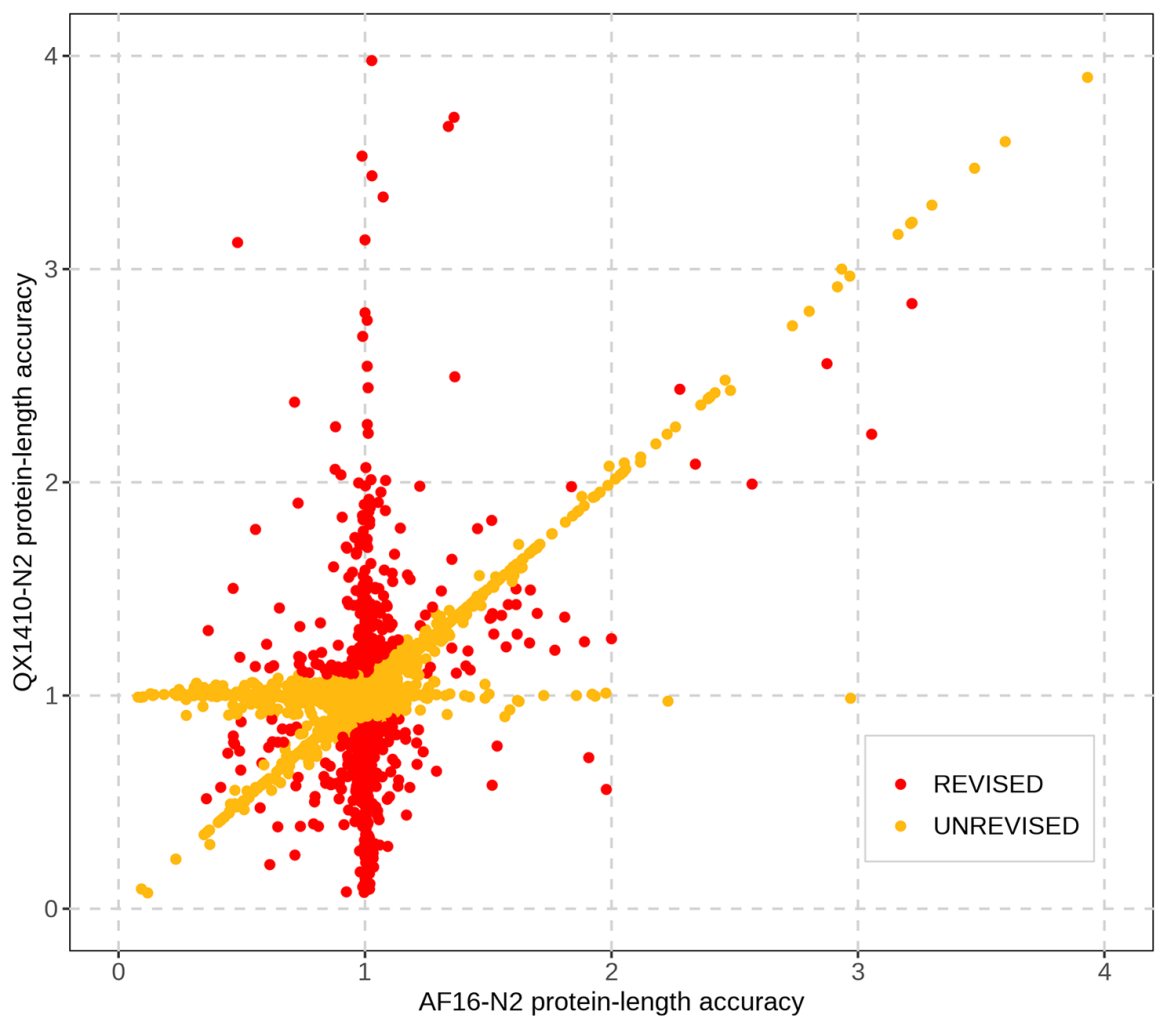
Protein-length differences from N2 between orthologous QX1410 and AF16 gene models. Each dot represents a gene in QX1410 that shares an N2 ortholog with an AF16 gene based on reciprocal BLAST analysis. We compared the protein length of each QX1410 and AF16 gene relative to its shared N2 ortholog and identified every QX1410 gene that is at least 10% shorter or longer in protein length (highlighted in red). We revised the RNA evidence used to model each gene that deviated in protein length relative to AF16 and made corrections when appropriate. Genes with protein-length accuracy values near to one (near-identical to the N2 gene) or genes that were near-identical in protein length between AF16 and QX1410 were not revised (highlighted in orange)

The vast majority of QX1410 genes are highly concordant in protein length with orthologous AF16 genes (Fig. 6). Specifically, a total of 13,464 genes share the same N2 ortholog in the QX1410 and AF16 strains based on reciprocal BLAST analysis, and 12,253 (91%) are near-identical in protein length between the two *C. briggsae* strains. Considering the differences in genome assemblies and gene modeling methods between QX1410 and AF16, protein sequences that are distant from the N2 strain but highly concordant between both *C. briggsae* strains are likely to define true differences in protein sequence evolution between the two species. However, the curated QX1410 gene set lags behind the AF16 gene models in protein-length accuracy, with QX1410 showing slightly lower counts for identical and 5% off reciprocal BLAST matches to the N2 gene models (Fig. 4). To assess the predictive power and usefulness of standalone transcriptomic data in generating gene models for newly assembled genomes, our gene predictions and manual curations were performed agnostic of homology to *C. elegans* gene model data. The discrepancy in protein-length accuracy between the QX1410 and AF16 genomes is therefore partly explained by the use of *C. elegans* homology data in the curation process of the AF16 gene models. For example, we found that the selection of the translation initiation site when multiple in-frame start codons were present in the first exon can underlie small differences in protein length between the QX1410 and AF16 genomes (Additional file 2, Fig. S2). As a result, the use of defined *C. elegans* start codons during the development of AF16 gene models contributes to the difference in identical reciprocal BLAST matches when compared to QX1410 gene models with computationally predicted open-reading frames. To assess alternative explanations for the discrepancy in protein length accuracy between AF16 and QX1410, we revised 755 QX1410 genes that were shorter or longer in protein length than the respective N2 and AF16 orthologs (Fig. 6). Based on RNA alignments alone, we made corrections to the coding sequences of 128 genes, leading to minor improvements in protein-length accuracy (Additional file 2, Table S1). These minor improvements suggest that curators missed potential repairs during our initial manual curation pass, which explains in part the lower protein-length accuracy observed in QX1410 gene models. The 627 genes that remained unchanged were in agreement with underlying RNA alignments or had insufficient RNA coverage to manually curate. Considering that the vast majority (78.6%) of QX1410 genes that were at least 10% shorter or longer in protein length relative to N2 protein lengths were expressed below 20 transcripts per million (TPM), the differences in protein-length accuracy between the AF16 and QX1410 gene models can be partly attributed to insufficient RNA sequencing depth necessary to resolve the correct structure of infrequently expressed genes (Additional file 2, Fig. S3). Moreover, our cDNA library preparation protocols did not employ any methods to specifically target or improve the yield of 5’ end mRNA sequences. As a result, the integrity of 5’ termini of our QX1410 gene models, and subsequently amino acid sequence near N-termini, was negatively affected by the underrepresented 5’ end sequences in our cDNA libraries. This limitation particularly affected genes that were initially predicted and curated solely from short-read RNA-seq, where we prepared cDNA libraries from polyA-selected mRNAs. Although the effects of 3’ end sequencing bias in protein-length accuracy can be minor at loci with high sequencing depth, many 5’ termini might remain incomplete, especially in gene models with low coverage that could not be manually curated.

**Table 1:** Gene and transcript summary

	N2 (WS280)	AF16 (WS280)	QX1410 (Automated)	QX1410 (Curated)
Total protein-coding genes	19,997	20,821	19,868	22,189
Total protein-coding transcripts	31,768	24,859	28,228	32,278

Gene and transcript count summary of automated and manually curated QX1410 gene models, AF16 gene models, and N2 gene models.

### Orthology analysis between ***C. briggsae*** and ***C. elegans*** shows incorrect gene duplications resolved in the QX1410 genome 

In parallel to the protein-length accuracy analysis based on orthologs identified using sequence similarity estimates, we employed OrthoFinder to study more complex orthologous relationships between *C. briggsae* and *C. elegans* genes [20]. This approach accounted for variable rates of sequence evolution between genes and facilitated the distinction of single-copy and multiple-copy orthologs between the two species. This distinction enabled the exclusion of multiple-copy orthologs, which prevented incorrect comparisons of protein length between genes of the same gene family. This approach also enabled the identification of orthologous relationships with lower identity that are missed using sequence similarity estimates used by BLAST search. We clustered 88,905 protein sequences translated from the gene models of the *C. elegans* N2 strain and both *C. briggsae* strains, QX1410 and AF16, into 17,790 putative orthologous groups. We identified 10,984 single-copy orthologs among all three strains, from which 86.2% were in agreement with reciprocal matches from the previous BLAST analysis, 0.3% were in disagreement, and 13.5% were not previously identified. Analysis of protein-length accuracy using only single-copy orthologs shows that the AF16 gene models still have higher counts of protein sequences with identical length to their N2 orthologs, but QX1410 gene models have higher counts for sequences that are within 5% of the protein length of their N2 orthologs (Additional file 2, Fig. S4, Table S2). The discrepancy in number of identical BLAST matches is explained by the same factors identified in the sequence similarity analysis, including the use of *C. elegans* homology data for AF16 or limitations in our sequencing depth and transcript coverage. Additionally, the exclusion of multiple-copy orthologs and the inclusion of previously missed orthologs influenced the change in pattern for sequences that are within 5% of the protein length of their N2 orthologs. Both QX1410 and AF16 gene models appear to have small subsets of genes with higher protein-length accuracy, which is reflected in the differences in protein-length accuracy counts when using different ortholog selection criteria (Fig. 6). Nonetheless, the orthology comparisons using either BLAST or OrthoFinder are largely in agreement and suggest that this manual curation of the QX1410 gene models using a single instance of transcriptome sampling provides gene models that are similar to the quality of the extensively curated AF16 gene models.

**Fig. 7:**
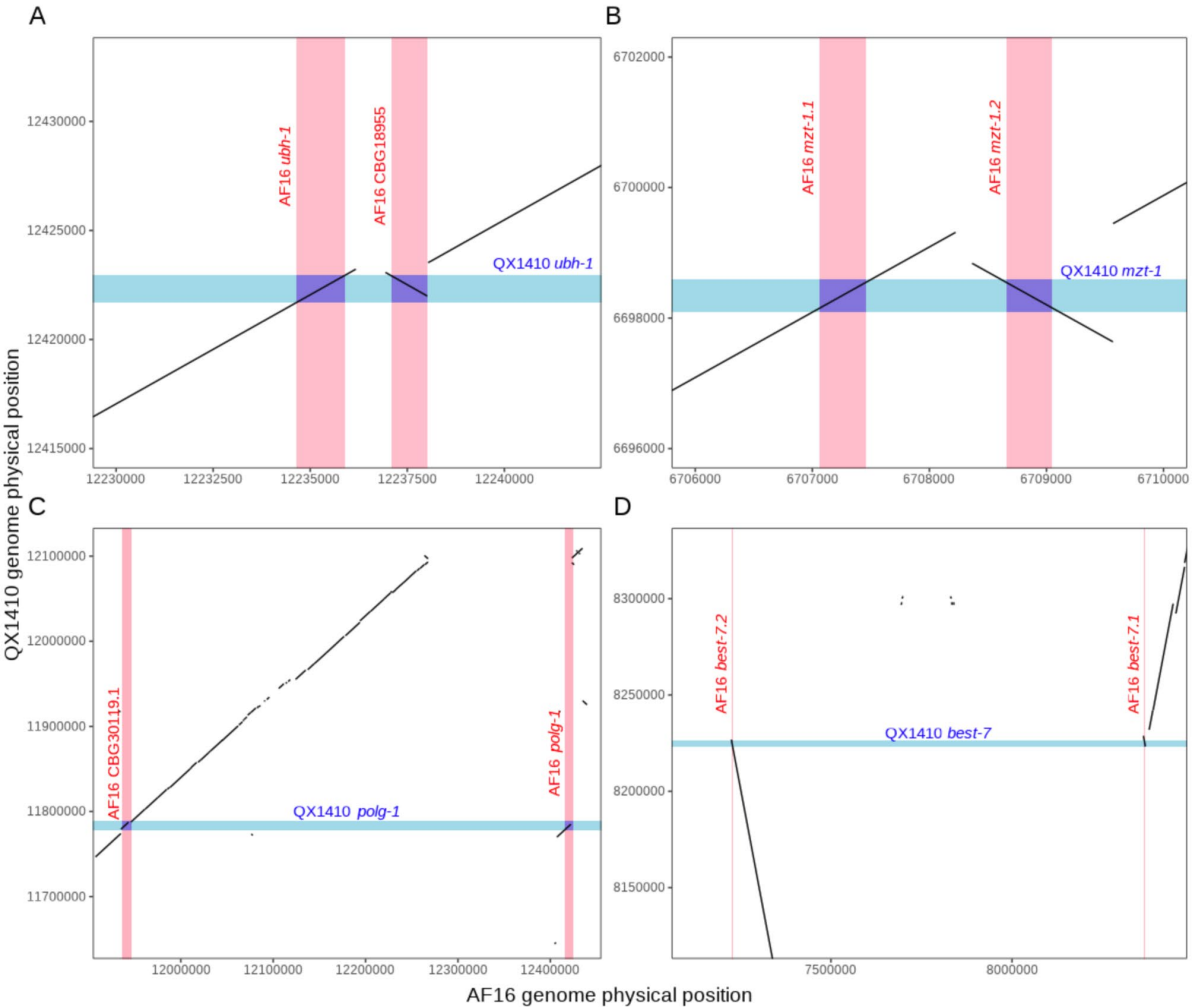
Examples of duplicated, inverted, and misplaced sequences in the AF16 genome assembly. Four plots showing the aligned QX1410 and AF16 genomes restricted to the physical positions of the *ubh-1* locus on chromosome V (**A**), *mzt-1* locus on chromosome I (**B**), *polg-1* locus on chromosome II (**C**), and *best-7* locus on chromosome IV (**D**). The gene boundaries are shaded in blue for the QX1410 genome, and shaded in red for the AF16 genome. The genomic sequences that contain each ortholog copy in the AF16 genome align uniquely to a single sequence in the QX1410 genome, demonstrating that the AF16 genome has an incorrect duplication. We selected two regions where the sequences are duplicated and in close proximity (**A** and **B**), and two regions where the sequences are duplicated and distant (**C** and **D**)

Interestingly, we identified a set of 317 single-copy orthologs between the QX1410 and N2 strains that were found as two or more copies in the AF16 strain. Manual inspection of orthologs that were found as two copies in AF16, but as single-copy in QX1410 and N2 (2:1:1 orthologs), suggests that additional gene copies originated from misassembly of genomic contigs in the AF16 genome leading to spurious duplications. Approximately 30% of 2:1:1 orthologs were found in two separate scaffolds in the AF16 genome, often in both chromosomal and non-chromosomal (unplaced) scaffolds. The remaining 70% of 2:1:1 orthologs were found in the same chromosomal scaffold and provided clear evidence of spurious duplications and inversions that occurred during genome assembly. For example, we found two orthologs of the N2 *ubh-1* gene on chromosome II of the AF16 genome: *Cbr-ubh-1* and *CBG18955*. We performed a collinearity analysis of the QX1410 and AF16 genomes and discovered that both AF16 *ubh-1* orthologs and adjacent sequences aligned uniquely with a single segment of the QX1410 genome. Additionally, the *CBG18955* locus appeared to be inverted relative to the *Cbr-ubh-1* locus (Fig. 7A). The same duplication pattern was observed in many other identified 2:1:1 orthologs, with or without inversion (Fig. 7B-D). By using the alignment patterns observed in the manually inspected QX1410 and AF16 2:1:1 orthologs, we identified 5,316 spurious intra-chromosomal duplication events in the AF16 genome affecting a total of 1,896 genes (Additional file 2, Table S3). Therefore, alignments between the AF16 and QX1410 genomes suggest that additional gene copies in the AF16 genome arose from artifactual duplications and/or inverted regions both within the chromosomal scaffolds and in unplaced scaffolds. Considering that the described genome assembly issues in the AF16 genome also affected multiple-copy orthologs, non-orthologous genes, and duplication events between chromosomes that were not included in this analysis, the QX1410 genome and gene models have not only resolved the duplication of numerous single-copy orthologs but also artifactual expansions of gene families present in the AF16 genome.

## Discussion

### The gap between ***C. elegans*** and ***C. briggsae*** genome resources

For several decades, *C. elegans* has served as a keystone animal model to study virtually all fields of biology. The experimental tractability of *C. elegans*, combined with the extensive community efforts to develop and improve its genome resources, makes it a highly versatile platform for biological research. Recently, advancements in sequencing and chromosomal scaffolding technologies have enabled the development of fast and effective methods to generate highly contiguous and complete genome assemblies for newly sequenced organisms [21]. Using these methods, improved reference genomes have been generated for numerous nematode species [3, 4, 22, 23]. These technologies have also been exploited to resolve structural errors, fill gaps, and repair collapsed repetitive regions in the *C. elegans* genome, yielding definitively one of the best metazoan genomes to date [24–26]. In parallel, *C. briggsae* has been used as a satellite model for comparative studies against *C. elegans*, providing new understanding of genetics, development, and evolution. Despite its usefulness, the quality and completeness of *C. briggsae* genome resources has lagged behind *C. elegans*. The publicly available version of the *C. briggsae* reference genome for the laboratory strain AF16 (referred to as ‘cb4’) contains thousands of unresolved gaps and hundreds of mis-scaffolded or unplaced sequences [5, 11, 12]. Although a recent attempt was made to repair these spurious rearrangements and gaps, the improved version (referred to as ‘cb4_improved’) is not available in public databases and the majority of gaps and unplaced sequences remained unresolved [12, 14]. Moreover, the problems with the AF16 genome are also detrimental to our ability to model protein-coding genes. Transcriptome and proteome sequences used to identify and model protein-coding genes fail to align in regions overlapping or near gaps and mis-scaffolded genomic sequences. As a result, hundreds of protein-coding genes in the AF16 reference genome remain incomplete, truncated, or misplaced. Although the structural integrity of the genes that are not proximal to genome assembly artifacts was largely unaffected, the physical coordinates of such genes were dramatically altered by genome assembly errors. These coordinate alterations obfuscate our measurements of linkage, heritability, and genome-wide association. Without drastic improvements to the *C. briggsae* genome resources, studies that use this species remain limited.

### A bridge between ***C. elegans*** and ***C. briggsae*** genome resources

In a previous study, we presented a new reference genome for the *Caenorhabditis briggsae* strain QX1410 [5]. Our transition to QX1410 was motivated by the uncertainty of the lineage of the AF16 population and potential alleles derived from the extensive laboratory passage of AF16, both problems that have been documented in the *C. elegans* laboratory strain N2 [26–29, 36]. The new QX1410 genome has only seven gaps and no unplaced sequences, resolving hundreds of mis-scaffolded sequences that were present in the existing AF16 genome. Additionally, we modeled protein-coding genes using a combination of short- and long-read transcriptome sequencing with modern gene prediction tools [5]. However, software-derived protein-coding gene prediction accuracy remains a challenge [30, 31]. When manually compared to transcriptome sequence alignments, software-derived gene models in the QX1410 genome have apparent errors such as the retention of non-coding sequences, incomplete coding sequences, incorrect intron-exon junctions, and gene fusions or splits, among others. Although new computational tools could identify and resolve these prediction errors in the near future, community-based manual curation has proven an effective method to detect errors and improve the quality of protein-coding gene models in nematodes [22, 32, 33]. Manual curation has been an essential step in the development of accurate and complete models for many nematode species, including *C. elegans* and *C. briggsae* [34]. Although whole-genome manual curation has been seldom used outside large-scale genome projects, modern gene annotation tools, such as the Apollo annotator platform, now provide a seamless environment for small, community-driven manual curation projects [15]. In this study, we assembled a team of nine high school, undergraduate, and graduate students to manually curate over 21,000 *C. briggsae* QX1410 genes, attesting to the feasibility of whole-genome community curation projects. We produced detailed workflows to identify and repair the most common errors in protein-coding gene predictions by leveraging both short- and long-read transcriptome alignments (Additional File 1). These workflows describe the interplay between gene model elements and transcriptomic signatures that were used to detect gene modeling errors. We believe these workflows can provide a foundation to engineer software and automate the detection of gene modeling errors, largely reducing the number of candidates considered for manual inspection in future curation projects. Unfortunately, the noisy nature of transcriptomic data paired with the unique differences in transcript expression between genes, makes the automated repair of such errors a rather unattainable task, a limitation that is reflected in the inconsistent precision of modern gene prediction software. We exploited the high level of protein length conservation between *C. briggsae* and *C. elegans* to quantify the changes in protein-length accuracy of manually curated QX1410 gene models relative to orthologous *C. elegans* N2 gene models. This analysis revealed substantial improvements in protein-length accuracy after manual curation, accompanied by a slight improvement in BUSCO completeness.

### Comparison of protein-length accuracy between QX1410 and AF16 gene models

We compared the protein-length accuracy results of both QX1410 and AF16 gene models, which revealed that fewer QX1410 gene models have reciprocal BLAST matches with identical or near-identical protein length to *C. elegans*. Although we identified several gene models with errors that were missed by curators, manual revisions of loci that underperform in protein-length accuracy in QX1410 revealed that most genes with shorter or longer protein sequences than their *C. elegans* orthologs have insufficient RNA coverage to manually curate. The RNA extraction protocols that we used attempt to maximize transcript representation across all stages of development, but it is possible that transcripts from certain stages or males are underrepresented in the pool of RNA extracted from our mixed-stage samples. It is also possible that even in genes with sufficient RNA coverage to be manually curated, our data only captured a subset of isoforms that might be shorter or longer than the isoform that was selected as a best reciprocal BLAST match between N2 and AF16. Such outcome could lead to minor inaccuracies in protein length accuracy between AF16 and QX1410 gene models. Additional transcriptome sequencing of stage- and sex-specific samples will be required to further maximize transcript discovery and improve the accuracy of gene models that currently lack sufficient RNA coverage. With single-cell RNA-seq becoming more affordable, future efforts to further improve the *C. briggsae* protein-coding gene models will also benefit from RNA sequencing of specific tissues that might be underrepresented in whole-animal transcriptome sequencing efforts. We also hypothesized that our cDNA library preparation methods led to underrepresented 5’ ends in our transcriptome alignments, an effect that we predict to exacerbate inaccuracies in genes modeled from infrequently expressed transcripts. The use of end-to-end RNA sequencing protocols or other 5’-end determination techniques will be needed to resolve the 5’ termini of gene models that underperform in protein-length accuracy. Better definition of the 5’ end of transcripts could also help define translation initiation sites that are currently arbitrarily defined by open reading frame prediction software in gene models with two or more in-frame start codons. Despite these limitations in library preparation and sequencing coverage, this single instance of manual curation led to QX1410 gene models that are comparable to AF16 gene models in protein-length accuracy and marginally improved in BUSCO completeness.

Our results demonstrated that AF16 gene models generally outperform QX1410 gene models in protein-length accuracy, and we explored how the limitations in our experimental design might explain this outcome. However, we also identified over 1,800 genes affected by spurious intra-chromosomal duplications stemming from genome assembly errors in the AF16 genome that are now resolved in the QX1410 genome. We demonstrated how these spurious duplications led to artifactual duplications of single-copy orthologs, altered orthologous relationships between species, and potential artifactual gene family expansions in AF16. The small differences in protein-length accuracy among the QX1410 and AF16 gene models are overshadowed by the numerous structural errors that persist in the AF16 genome assembly. Although we have documented a list of problematic genes for future reference, the continued use of the AF16 reference resources in its current state will only further propagate these errors.

### A future prospect for ***Caenorhabditis briggsae***

Presently, the problems described with the AF16 genome and the uncertainty of the AF16 strain fidelity pose major limitations for the use of *C. briggsae* in genomic studies. Until these fundamental issues in AF16 are resolved, the high level of sequence contiguity in the QX1410 genome provides a reliable platform to continue to improve and expand the *C. briggsae* genome resources. The availability of high-quality *C. briggsae* reference gene models in the correct genomic context will also help establish complete and accurate gene family relationships within and between species, improve measurements of genetic relatedness among *C. briggsae* wild isolates and phylogeographic groups, and enable candidate gene discovery in genome-wide association studies. Another major motivation to improve the quality of *C. briggsae* genome resources is to enable new avenues to investigate *Caenorhabditis* genome evolution. Specifically, recent studies in *C. elegans* wild isolates have led to the discovery of punctuated genomic regions with abnormally high genetic diversity relative to expectations of diversity in self-fertilizing organisms. These hyper-divergent regions are thought to retain ancestral alleles maintained by balancing selection during the evolutionary history of *C. elegans*. These regions appear to be implicated in local adaptation because they harbor unique sets of environmental response genes among different *C. elegans* wild isolates [35]. The characterization of the gene content of hyper-divergent regions in *C. elegans* was made possible because of the high-quality genome resources available for this species. Interestingly, evidence points to the presence of hyper-divergent regions in a small subset of *C. briggsae* wild strains [35]. The availability of complete and accurate genome resources for *C. briggsae* will be essential to characterize and test hypotheses surrounding the origin and function of these regions.

## Conclusions

In this study, we presented a detailed workflow to manually curate gene models using short- and long-read RNA sequencing data. Our manual curation efforts led to improved gene models for the newly sequenced reference genome for the *C. briggsae* strain QX1410, attesting to the effectiveness of community-based manual curation. We showed that comparative genomic analysis using a related species with high-quality reference genome(s) and gene models can be used to quantify improvements in gene model quality in a newly sequenced genome. According to our metric based on protein sequence length similarity between *C. elegans* and *C. briggsae* orthologs, the manually curated QX1410 gene models are similar in quality to the extensively curated gene models of the laboratory strain AF16. Considering the pervasive errors in the AF16 genome assembly, the use of QX1410 genome resources provides a reliable and improved platform to perform genomic studies in this species and make comparisons against other nematode species.

## Materials and methods

### Nematode culture

Animals were reared at 20 °C on standard nematode growth medium (NGM) plates and *Escherichia coli* OP50 was used as a food source.

### RNA library preparation

We extracted RNA from a mixed-stage and mixed-sex population composed of samples from all larval stages, adults, dauers, and males. Plates containing adults and larvae from every stage were prepared by chunking every two days for several generations. Plates containing dauer and arrested L1 and L2 larvae were prepared by allowing the plate to starve. Male-enriched plates were prepared by setting up crosses between male and hermaphrodite animals and expanding the population for up to three generations. We collected a sample from one 10 cm plate for each population (mixed-stage, starved, male-enriched) into 100ul S Basal. Samples were flash frozen in liquid nitrogen and stored at -80 °C. RNA was extracted from each sample using 1 mL TRIzol reagent (Invitrogen, catalog no. 15,596,026) with addition of 100 uL of acid-washed sand to aid sample homogenization, and resuspended in nuclease-free water. We used a Nanodrop spectrophotometer (ThermoFisher) to quantify the purity of the RNA samples, and a Bioanalyzer and a Qubit (ThermoFisher) were used to determine RNA concentration. The Qubit RNA HS Assay kit (ThermoFisher, catalog no. Q32852) was used. We pooled 1.5 mg RNA from each sample, and purified and concentrated the pooled RNA using the RNeasy MinElute Cleanup kit (Qiagen, catalog no. 74,024). We eluted the purified RNA into nuclease-free water and repeated the quality control steps (purity and concentration determination) as described previously.

### Short-read RNA sequencing

The Illumina RNA-seq library was prepared in a 96-well plate. We purified and enriched mRNA from 1 ug of total RNA using the NEBNext Poly(A) Magnetic Isolation Module (New England Biolabs, catalog no. E7490L). RNA fragmentation, first and second strand cDNA synthesis, and end-repair processing was performed using the NEBNext Ultra II RNA Library Prep with Sample Purification Beads (New England Biolabs, catalog no. E7775L). We ligated adapters in the cDNA library using adapters and unique dual indexes from the NEBNext Multiplex Oligos for Illumina (New England Biolabs, catalog no. E6440L). All procedures were performed following the manufacturer protocols. We used Qubit dsDNA BR Assay Kit (Invitrogen, catalog no. Q32853) to determine the concentration of the RNA library. The library was then pooled and qualified using the 2100 Bioanalyzer (Agilent) at Novogene, CA, USA. We sequenced the pooled library with the Illumina NovaSeq 6000 platform (150-bp paired-end reads).

### Long-read RNA sequencing

The PacBio Iso-Seq full-length sequencing library was prepared using 300 ng of total RNA using NEBNext Single Cell/Low Input cDNA Synthesis and Amplification Module (NEB, catalog no. E6421) and SMRTbell Express Template Prep Kit 2.0 (Pacific Biosciences, catalog no. 100-938-900). This library was prepared in the Duke Center for Genomic and Computational Biology’s Sequencing Technologies Core facility, and was sequenced using three SMRT cells.

### Repeat masking

Prior to gene prediction, we masked repetitive sequences in the QX1410 genome to avoid spurious predictions. We generated a custom repeat library using a previously described approach [3, 37]. In summary, we used RepeatModeler from RepeatMasker v2.0.1 [38] for *de novo* repetitive sequence identification. Additionally, we identified transposable elements using Transposon PSI [39], and long terminal repeat (LTR) retrotransposons using LTR harvest from GenomeTools v1.6.1 [40, 41]. Identified LTR retrotransposons were annotated using LTRdigest from GenomeTools with HMM domain profiles from Gypsy Database 2.0 [42] and select Pfam domains [43] (listed in tables SB1 and SB2 of [44]). We removed repeat candidate sequences without conserved protein domains using *gt-select* from GenomeTools. Lastly, we retrieved Rhabditida-specific repeats Dfam [45] and *C. elegans* ancestral repeats from RepBase [46]. We merged newly generated and retrieved repeat libraries into a single redundant library. We clustered and classified repeats in the redundant library using VSEARCH v2.14.2 [47] and RepeatClassifier from RepeatMasker, respectively. Unclassified repeats with significant BLAST hits to the *C. elegans* proteome (WS279) were removed. We soft-masked the QX1410 genome assembly using RepeatMasker.

### Automated protein-coding gene prediction

The QX1410 genome was retrieved from NCBI under the accession PRJNA784955 [5]. We aligned short-read RNA sequences to the soft-masked QX1410 genome using STAR v2.7.3a [48] with a maximum intron size of 10 kilobases, and generated protein-coding gene predictions using BRAKER v2.1.6 [19]. Additionally, we generated high-quality transcripts from PacBio long RNA reads using isoseq3 v3.4.0 [16] and aligned them to the QX1410 genome using minimap2 v2.26-r1175 [49]. We performed transcriptome assembly using StringTie v2.1.2 [17] from PacBio high-quality transcript alignments. We predicted coding sequences in the assembled transcripts using TransDecoder v5.5.0 [50]. We assessed the biological completeness of generated gene models using BUSCO v5.0 [51].

### Manual curation of gene prediction errors

Short- and long-read sequence alignments and gene models were uploaded as individual tracks to the Apollo platform v2.6.4 [15] for manual curation. We repaired three classes of gene prediction errors: gene splits, gene fusions, and intron chain errors (additional, missing, or modified exons). We identified potential gene splits by inspecting RNA alignments in intergenic regions. When two adjacent gene models have uninterrupted long- and short-read RNA coverage across their intergenic region, we merged both models and re-predicted the coding sequence. If the gene merger led to a premature stop codon, we reverted the merger. We identified gene fusions by searching for gaps in RNA coverage within a single gene accompanied by a predicted intron that bypasses a stop codon with RNA coverage. Additional, missing, or incorrect exons of a gene were repaired using the consensus exon-intron structure between long-read transcripts and collapsed short-read alignments. If long-read RNA transcripts were not available to confirm a potential split, fusion, incorrect exon identified solely by short-read RNA reads, or were in disagreement with short-read RNA alignments, we kept both alternative models (original and manually curated). We did not attempt any manual edits to gene models with less than 2x Illumina coverage. Gene models below this threshold were preserved as they were initially predicted by software.

### Manual curation of coding sequences and untranslated regions

We used the built-in open-reading frame (ORF) prediction tool from Apollo to select the optimal coding sequence for each transcript. This tool weights longer ORFs over coding sequence continuity across all exons. We manually set start and stop codons that led into a shorter ORF that spanned a higher number of exons compared to the predicted ORF with longer coding sequence. We exploited full-length, long-read transcripts to extend untranslated regions of every gene. When long-read transcripts were unavailable, we used the longest set of short read alignments that overlapped with the coding sequence of the gene. We matched the UTR boundaries of gene isoforms with identical terminal exons. We did not annotate UTRs of genes in close proximity and in the same strand with continuous short-read coverage throughout their intergenic region.

### Manual curation of additional isoforms and new genes

In many cases, multiple isoforms were predicted by BRAKER and StringTie. We removed any isoforms that were unsupported by both short- and long-read alignments. We added any potential isoforms that were present as structurally unique long-read transcripts. Isoforms modeled from long-read transcripts that had substantially shorter coding sequences (one or more non-coding exons) were kept. Differences in structure observed between the predicted gene models and a subset of the short-read alignments were also included as additional isoforms. In cases where multiple structural differences were observed in short-read alignments, we modeled all possible structural permutations that had a continuous ORF from the first to the last exon. When a multi-exon structure was observed in the short-read alignments and no genes were predicted at that locus, we modeled a gene *de novo*. We discarded any *de novo* gene models that did not have an ORF.

### Protein sequence length analysis

We generated BLAST v5 libraries for each set of *C. briggsae* (QX1410 and AF16) protein sequences and for *C. elegans* (N2). We performed forward and backward BLASTp v2.12 searches between N2 and each *C. briggsae* strain [52]. We selected the best hit (ranked using expectation value and bitscore) for every protein sequence in the forward search that had a reciprocal best hit in the backward search. We calculated the ratio of protein sequence length between each *C. briggsae* protein sequence and its reciprocal *C. elegans* protein sequence. We kept only one reciprocal hit per gene. We counted the number of protein sequences with identical protein length matches and matches that were within 5% of the length of its respective reciprocal hit.

### Orthology analysis

We extracted protein sequences from gene model files in GFF format using Gffread v0.12.1 [53]. The GFF files were filtered to only keep the longest isoform per gene using *agat_sp_keep_longest_isoform.pl* from AGAT v0.8.1 [54]. We clustered protein sequences extracted from *C. briggsae* QX1410, *C. briggsae* AF16, and *C. elegans* N2 gene models into orthologous groups using OrthoFinder v2.1.4. We classified single-copy and multiple copy orthologs across all three strains in R. We performed the same protein sequence length analysis described above using single-copy orthologs identified by OrthoFinder across all three strains (AF16, QX1410, and N2).

### Collinearity analysis

The AF16 (WS280) and QX1410 genomes were aligned using NUCleotide MUMmer (NUCmer) v3.1, allowing a maximum gap of 500 bp [55]. Duplicated sequences of the AF16 genomes were identified using R v4.1.1 by selecting alignments that had a single set of coordinates in the QX1410 genome, but distinct sets of coordinates in the AF16 genome.

### Electronic supplementary material

Below is the link to the electronic supplementary material.

Supplementary Material 1

Supplementary Material 2

## Data Availability

The datasets (raw sequencing data, genome assembly, and gene model files) supporting the conclusions of this article are available in NCBI under the study accession PRJNA784955. Code used to produce analyses and figures are available in GitHub at https://github.com/AndersenLab/briggsae_gene_models_MS.

## References

[1] Kanzaki N, Tsai IJ, Tanaka R, Hunt VL, Liu D, Tsuyama K (2018). Biology and genome of a newly discovered sibling species of *Caenorhabditis elegans*. Nat Commun.

[2] Stevens L, Félix M-A, Beltran T, Braendle C, Caurcel C, Fausett S (2019). Comparative genomics of 10 new *Caenorhabditis* species. Evol Lett.

[3] Teterina AA, Willis JH, Phillips PC (2020). Chromosome-level assembly of the *Caenorhabditis remanei* Genome reveals conserved patterns of Nematode Genome Organization. Genetics.

[4] Noble LM, Yuen J, Stevens L, Moya N, Persaud R, Moscatelli M et al. Selfing is the safest sex for *Caenorhabditis tropicalis*. Elife. 2021;10. 10.7554/eLife.62587 PMC7853720 33427200

[5] Stevens L, Moya ND, Tanny RE, Gibson SB, Tracey A, Na H et al. Chromosome-level reference genomes for two strains of *Caenorhabditis briggsae*: an Improved platform for comparative Genomics. Genome Biol Evol. 2022;14. 10.1093/gbe/evac042 PMC9011032 35348662

[6] Cutter AD, Félix M-A, Barrière A, Charlesworth D (2006). Patterns of nucleotide polymorphism distinguish temperate and tropical wild isolates of *Caenorhabditis briggsae*. Genetics.

[7] Félix M-A, Duveau F (2012). Population dynamics and habitat sharing of natural populations of *Caenorhabditis elegans* and *C. briggsae*. BMC Biol.

[8] Crombie TA, Zdraljevic S, Cook DE, Tanny RE, Brady SC, Wang Y et al. Deep sampling of hawaiian *Caenorhabditis elegans* reveals high genetic diversity and admixture with global populations. Elife. 2019;8. 10.7554/eLife.50465 PMC6927746 31793880

[9] Thomas CG, Wang W, Jovelin R, Ghosh R, Lomasko T, Trinh Q (2015). Full-genome evolutionary histories of selfing, splitting, and selection in *Caenorhabditis*. Genome Res.

[10] Stein LD, Bao Z, Blasiar D, Blumenthal T, Brent MR, Chen N (2003). The genome sequence of *Caenorhabditis briggsae*: a platform for comparative genomics. PLoS Biol.

[11] Ross JA, Koboldt DC, Staisch JE, Chamberlin HM, Gupta BP, Miller RD (2011). *Caenorhabditis briggsae* recombinant inbred line genotypes reveal inter-strain incompatibility and the evolution of recombination. PLoS Genet.

[12] Ren X, Li R, Wei X, Bi Y, Ho VWS, Ding Q (2018). Genomic basis of recombination suppression in the hybrid between *Caenorhabditis briggsae* and *C. nigoni*. Nucleic Acids Res.

[13] Jhaveri N, van den Berg W, Hwang BJ, Muller H-M, Sternberg PW, Gupta BP. Genome annotation of *Caenorhabditis briggsae* by TEC-RED identifies new exons, paralogs, and conserved and novel operons. G3. 2022;12. 10.1093/g3journal/jkac101 PMC9258526 35485953

[14] Harris TW, Arnaboldi V, Cain S, Chan J, Chen WJ, Cho J (2020). WormBase: a modern Model Organism Information Resource. Nucleic Acids Res.

[15] Dunn NA, Unni DR, Diesh C, Munoz-Torres M, Harris NL, Yao E (2019). Apollo: democratizing genome annotation. PLoS Comput Biol.

[16] IsoSeq. IsoSeq3 - Scalable De Novo Isoform Discovery from Single-Molecule PacBio Reads. Github.

[17] Kovaka S, Zimin AV, Pertea GM, Razaghi R, Salzberg SL, Pertea M (2019). Transcriptome assembly from long-read RNA-seq alignments with StringTie2. Genome Biol.

[18] TransDecoder. : TransDecoder source. Github.

[19] Hoff KJ, Lomsadze A, Borodovsky M, Stanke M, Kollmar M (2019). Whole-genome annotation with BRAKER. Gene Prediction: methods and protocols.

[20] Emms DM, Kelly S (2019). OrthoFinder: phylogenetic orthology inference for comparative genomics. Genome Biol.

[21] Rhie A, McCarthy SA, Fedrigo O, Damas J, Formenti G, Koren S (2021). Towards complete and error-free genome assemblies of all vertebrate species. Nature.

[22] Doyle SR, Tracey A, Laing R, Holroyd N, Bartley D, Bazant W (2020). Genomic and transcriptomic variation defines the chromosome-scale assembly of *Haemonchus contortus*, a model gastrointestinal worm. Commun Biol.

[23] de la Gonzalez PM, Thomson M, Trivedi U, Tracey A, Tandonnet S, Blaxter M. A telomere-to-telomere assembly of *Oscheius tipulae* and the evolution of rhabditid nematode chromosomes. G3. 2021;11. 10.1093/g3journal/jkaa020 PMC8022731 33561231

[24] Hillier LW, Coulson A, Murray JI, Bao Z, Sulston JE, Waterston RH (2005). Genomics in *C. elegans*: so many genes, such a little worm. Genome Res.

[25] Tyson JR, O’Neil NJ, Jain M, Olsen HE, Hieter P, Snutch TP (2018). MinION-based long-read sequencing and assembly extends the *Caenorhabditis elegans* reference genome. Genome Res.

[26] Yoshimura J, Ichikawa K, Shoura MJ, Artiles KL, Gabdank I, Wahba L (2019). Recompleting the *Caenorhabditis elegans* genome. Genome Res.

[27] Gems D, Riddle DL (2000). Defining wild-type life span in *Caenorhabditis elegans*. J Gerontol A Biol Sci Med Sci.

[28] Vergara IA, Mah AK, Huang JC, Tarailo-Graovac M, Johnsen RC, Baillie DL (2009). Polymorphic segmental duplication in the nematode *Caenorhabditis elegans*. BMC Genomics.

[29] Sterken MG, Snoek LB, Kammenga JE, Andersen EC (2015). The laboratory domestication of *Caenorhabditis elegans*. Trends Genet.

[30] Hoff KJ, Lange S, Lomsadze A, Borodovsky M, Stanke M (2016). BRAKER1: unsupervised RNA-Seq-based genome annotation with GeneMark-ET and AUGUSTUS. Bioinformatics.

[31] Cook DE, Valle-Inclan JE, Pajoro A, Rovenich H, Thomma BPHJ, Faino L (2019). Long-read annotation: automated eukaryotic genome annotation based on Long-Read cDNA sequencing. Plant Physiol.

[32] Rödelsperger C, Athanasouli M, Lenuzzi M, Theska T, Sun S, Dardiry M (2019). Crowdsourcing and the feasibility of manual gene annotation: a pilot study in the nematode *Pristionchus pacificus*. Sci Rep.

[33] Athanasouli M, Witte H, Weiler C, Loschko T, Eberhardt G, Sommer RJ (2020). Comparative genomics and community curation further improve gene annotations in the nematode *Pristionchus pacificus*. BMC Genomics.

[34] Williams GW, Davis PA, Rogers AS, Bieri T, Ozersky P, Spieth J (2011). Methods and strategies for gene structure curation in WormBase. Database.

[35] Lee D, Zdraljevic S, Stevens L, Wang Y, Tanny RE, Crombie TA (2021). Balancing selection maintains hyper-divergent haplotypes in *Caenorhabditis elegans*. Nat Ecol Evol.

[36] Andersen EC, Bloom JS, Gerke JP, Kruglyak L (2014). A variant in the neuropeptide receptor npr-1 is a major determinant of *Caenorhabditis elegans* growth and physiology. PLoS Genet.

[37] Berriman M, Coghlan A, Tsai IJ. Creation of a comprehensive repeat library for a newly sequenced parasitic worm genome. 2018. 10.1038/protex.2018.054.

[38] Smit AFA, Hubley R, Green P. RepeatMasker Open-4.0. 2013–2015. 2015.

[39] TransposonPSI. : An Application of PSI-Blast to Mine (Retro-)Transposon ORF Homologies. http://transposonpsi.sourceforge.net/. Accessed 12 Oct 2020.

[40] Ellinghaus D, Kurtz S, Willhoeft U (2008). LTRharvest, an efficient and flexible software for de novo detection of LTR retrotransposons. BMC Bioinformatics.

[41] Gremme G, Steinbiss S, Kurtz S (2013). GenomeTools: a comprehensive software library for efficient processing of structured genome annotations. IEEE/ACM Trans Comput Biol Bioinform.

[42] Llorens C, Futami R, Covelli L, Domínguez-Escribá L, Viu JM, Tamarit D (2011). The Gypsy database (GyDB) of mobile genetic elements: release 2.0. Nucleic Acids Res.

[43] Finn RD, Bateman A, Clements J, Coggill P, Eberhardt RY, Eddy SR (2014). Pfam: the protein families database. Nucleic Acids Res.

[44] Steinbiss S, Willhoeft U, Gremme G, Kurtz S (2009). Fine-grained annotation and classification of de novo predicted LTR retrotransposons. Nucleic Acids Res.

[45] Hubley R, Finn RD, Clements J, Eddy SR, Jones TA, Bao W (2016). The Dfam database of repetitive DNA families. Nucleic Acids Res.

[46] Bao W, Kojima KK, Kohany O (2015). Repbase Update, a database of repetitive elements in eukaryotic genomes. Mob DNA.

[47] Rognes T, Flouri T, Nichols B, Quince C, Mahé F (2016). VSEARCH: a versatile open source tool for metagenomics. PeerJ.

[48] Dobin A, Davis CA, Schlesinger F, Drenkow J, Zaleski C, Jha S (2013). STAR: ultrafast universal RNA-seq aligner. Bioinformatics.

[49] Li H (2018). Minimap2: pairwise alignment for nucleotide sequences. Bioinformatics.

[50] TransDecoder, Wiki. Github.

[51] Simão FA, Waterhouse RM, Ioannidis P, Kriventseva EV, Zdobnov EM (2015). BUSCO: assessing genome assembly and annotation completeness with single-copy orthologs. Bioinformatics.

[52] Camacho C, Coulouris G, Avagyan V, Ma N, Papadopoulos J, Bealer K (2009). BLAST+: architecture and applications. BMC Bioinformatics.

[53] Pertea G, Pertea M. GFF Utilities: GffRead and GffCompare. F1000Res. 2020;9. 10.12688/f1000research.23297.1 PMC7222033 32489650

[54] Dainat J, Hereñú D, LucileSol. pascal-git. NBISweden/AGAT: AGAT-v0.8.1. 2022.

[55] Marçais G, Delcher AL, Phillippy AM, Coston R, Salzberg SL, Zimin A (2018). MUMmer4: a fast and versatile genome alignment system. PLoS Comput Biol.

