# Supplementary material for: Novel and improved Caenorhabditis briggsae gene models generated by community curation
Source: BMC Genomics. 2023 Aug 25;24:486. doi: 10.1186/s12864-023-09582-0 (PMC10463891; doi:10.1186/s12864-023-09582-0)
Supplement: Supplementary file 2 — Supplementary Material 2 [file 12864_2023_9582_MOESM2_ESM.pdf]

Novel and improved *Caenorhabditis briggsae* gene models generated by community curation

Nicolas D. Moya[1,2], Lewis Stevens[1,3], Isabella R. Miller[1], Chloe E. Sokol[1], Joseph L. Galindo[1], Alexandra D. Bardas[1], Edward S. H. Koh[1], Justine Rozenich[1], Cassia Yeo[1], Maryanne Xu[1], and Erik C. Andersen[1,‡]

1. Department of Molecular Biosciences, Northwestern University, Evanston, IL 60208, USA
2. Interdisciplinary Biological Sciences Program, Northwestern University, Evanston, IL 60208, USA
3. Tree of Life, Wellcome Sanger Institute, Cambridge, UK

Additional file 2

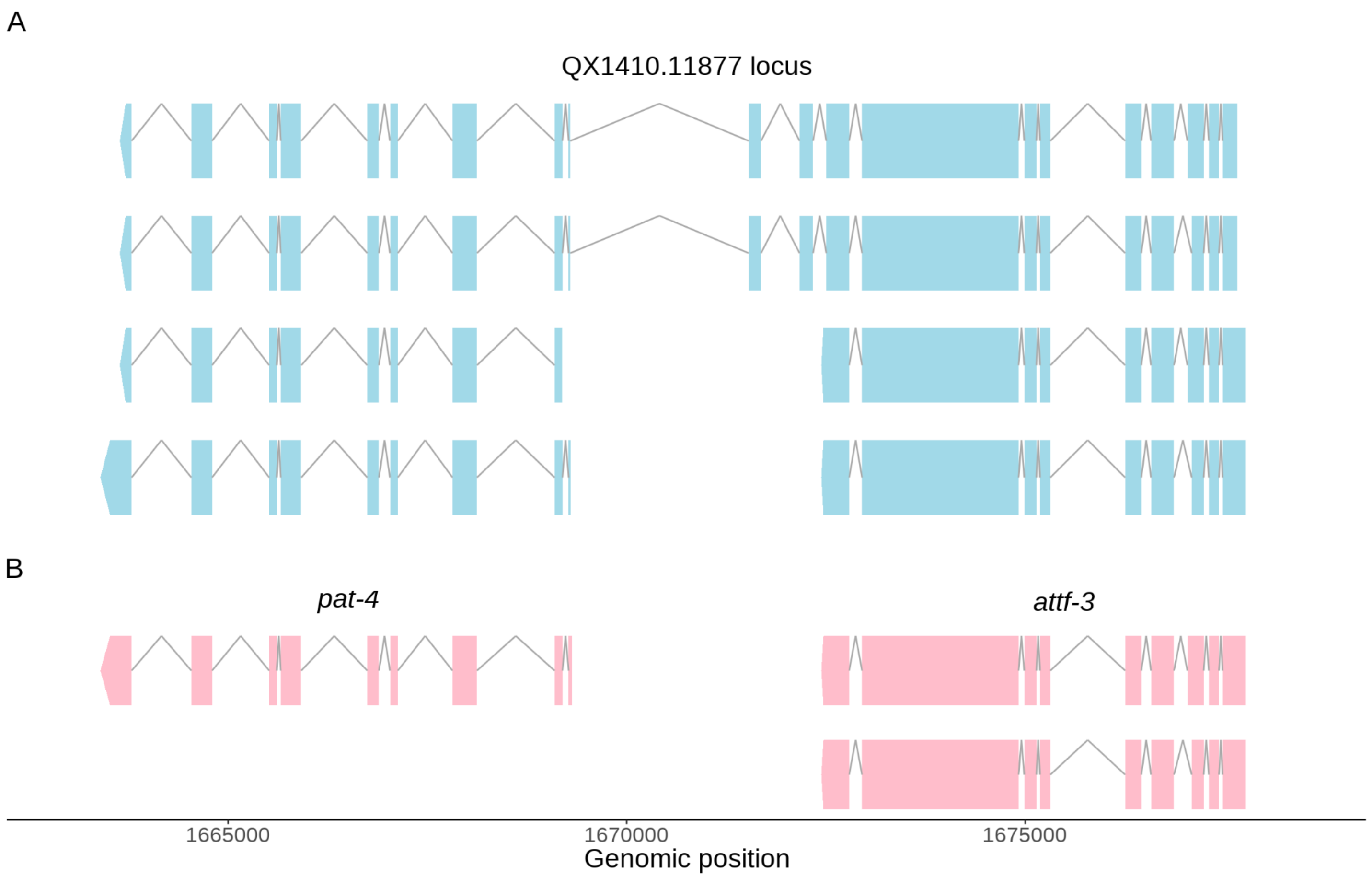

Figure S1: Example of BUSCO duplication reduction after manual curation
Plot showing the gene models located on chromosome III from 1,663,398 to 1,677,797 before (A) and after (B) curation. Six software-derived models are shown in blue and are annotated as isoforms of a single gene. Three spurious isoforms were removed during the curation process, and the locus was divided into two separate genes, orthologous to the *C. elegans pat-4* and *attf-3* genes. The *pat-4* gene is part of the BUSCO database, and it is no longer duplicated. The *attf-3* gene is composed of two isoforms that have differences in the 3' termini of exon 3.

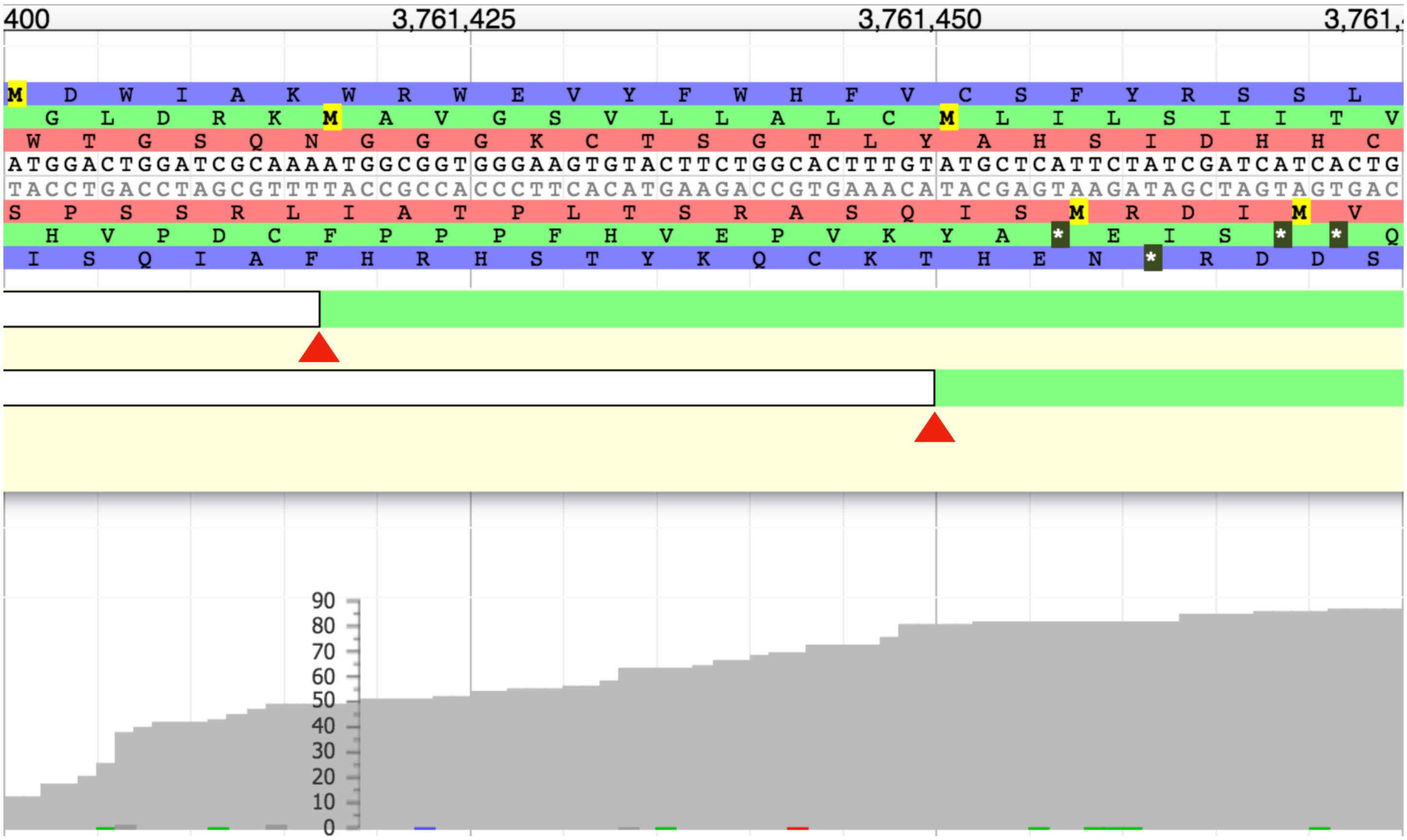

Figure S2: Example of alternative translation initiation site

Screen capture showing a genomic window of 75bp (located on chromosome III from 3,761,400 to 3,761,475) that covers the 5’ termini of the *gar-2 C. briggsae* ortholog. Two alternative in-frame translation initiation sites are highlighted with red arrows. Both sites have similar RNA coverage (shown in gray).

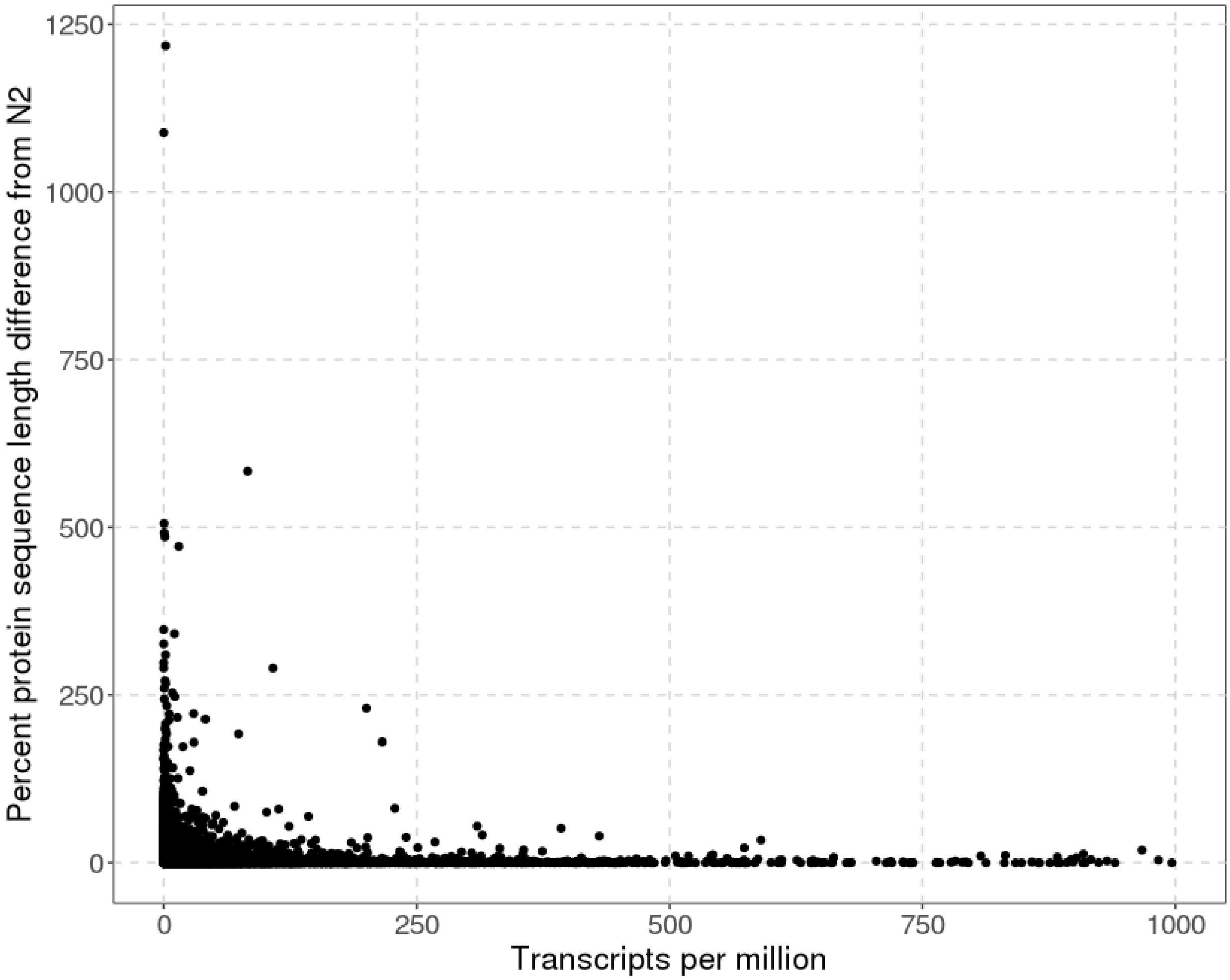

Figure S3: Protein-length accuracy of QX1410 genes by TPM
Plot showing the transcripts per million for each QX1410 gene against its percent protein length distance to its N2 ortholog.

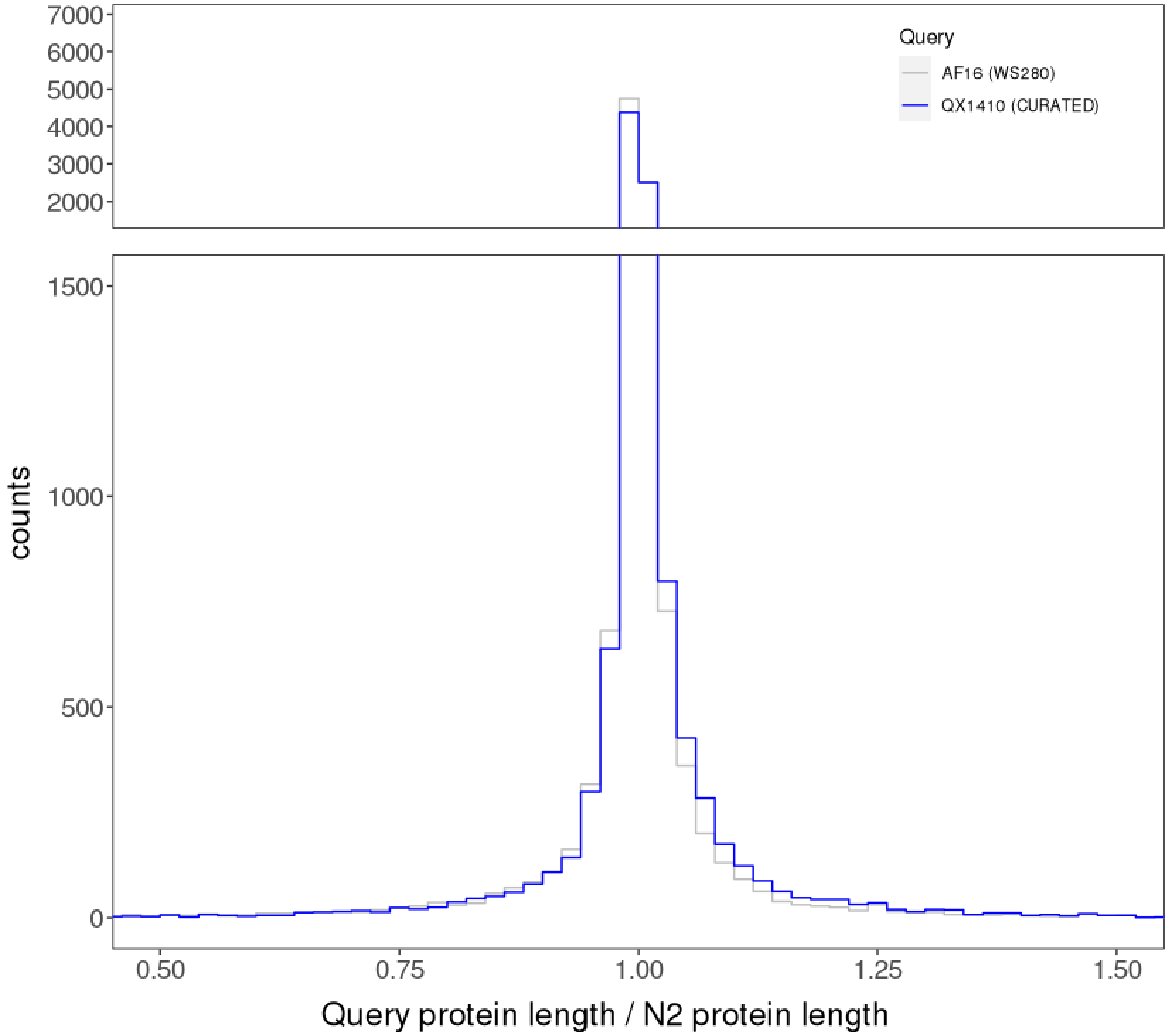

Figure S4: Protein length accuracy of *C. briggsae* gene models relative *C. elegans* N2 inferred single-copy orthologs

We calculated the protein length accuracy of both curated QX1410 gene models and AF16 (WS280) gene models using single-copy orthologs identified through phylogenetic orthology analysis. A ratio of one represents a *C. briggsae* gene with a protein length that perfectly matches its single-copy ortholog in N2.

Table S1: Protein-length accuracy and BUSCO completeness before and after revision of 755 loci with deviations in protein length

	Before revision	After revision
one-to-one matches to N2	2601	2710
5% off matches to N2	7849	7940
BUSCO completeness	99.7%	99.7%

Table S2: Protein-length accuracy using phylogeny-inferred orthologs between QX1410, AF16, and N2.

	AF16	QX1410
one-to-one matches to N2	2222	2173
5% off matches to N2	8762	8811

Table S3: Intra-chromosomal duplications in the AF16 genome and affected genes

segment A	segment B	identity	genes in segment A	genes in segment B
I:12895-14211	I:396029-397345	99.57	Cbr-rps-29	CBG11412
I:175947-178569	I:169983-172605	99.7	CBG11861, CBG00361, CBG11492	Cbr-arrd-2, Cbr-sbds-1, CBG11493
I:134401-138262	I:126814-130653	99.77	CBG01029, CBG15263, Cbr-ncs-3	Cbr-lim-6
I:124226-127163	I:131671-134750	99.42	CBG30931, Cbr-pfn-3	CBG15264
I:50373-56695	I:42341-48591	99.62	CBG01049, CBG01048, CBG01046, CBG11525, CBG11524	Cbr-glt-5, CBG00337, Cbr-fmo-4
I:1171622-1178466	I:1218277-1225147	96.33	CBG16333	CBG18556, CBG00846, CBG00845, Cbr-hpo-38, Cbr-acr-15, CBG16326
I:1352610-1357683	I:1361512-1366585	99.22	CBG00754, CBG27416	CBG15737, CBG27418
I:1463157-1465588	I:1466607-1469038	97.69	CBG00785	CBG31329, CBG00786
I:1678479-1681854	I:1700745-1704120	99.27	CBG25180	CBG30327
I:1807933-1810419	I:1811400-1813886	96.44	CBG30809	CBG22020, CBG08816
I:2001351-2004701	I:2006517-2009867	97.19	CBG21555, CBG21557, CBG31291	CBG21559
I:2006686-2009893	I:2001512-2004758	97.81	CBG21559	CBG21557, CBG31291
I:3219715-3226441	I:2424060-2430786	99.9	CBG29818, CBG11638, CBG17031, CBG30501, Cbr-tmem-218	Cbr-pqn-59.1, CBG30439, CBG26638
I:2797516-2798692	I:2802327-2803504	99.83	CBG22202	CBG22203
I:3208166-3215423	I:3275534-3282791	99.72	Cbr-smo-1, CBG22302, Cbr-glna-2, CBG26833	CBG15102, CBG15103, CBG15104, CBG13342, CBG10509, CBG10508
I:3456270-3466382	I:3456270-3466382	99.98	Cbr-xrep-4, Cbr-trpp-8, CBG14903, CBG00725, CBG24735, Cbr-pes-1, CBG10454, Cbr-gnrr-3	Cbr-xrep-4, Cbr-trpp-8, CBG14903, CBG00725, CBG24735, Cbr-pes-1, CBG10454, Cbr-gnrr-3
I:3560841-3566940	I:3567941-3574040	99.8	Cbr-elo-8, Cbr-srg-3, CBG17473	CBG30964, CBG22594, CBG10425, CBG01955
I:4963727-4964839	I:15222377-15223491	94.92	CBG03918	CBG17036
I:5594552-5601685	I:5606871-5614004	99.98	CBG03762, CBG03761, CBG03760, Cbr-fip-3.1, CBG18231, Cbr-exos-1, CBG17865, CBG30898	CBG03699, Cbr-cel-1, CBG17869, CBG17870
I:8006840-8008057	I:12076549-12077849	85.01	CBG31390	CBG00002
I:5779531-5781829	I:5830921-5833219	99.91	CBG30597, Cbr-ttr-31	CBG27553

I:1673321-1674689	I:13084727-13086094	99.78	CBG26161	CBG16717
I:6706546-6707749	I:6708365-6709568	99.83	Cbr-mzt-1.2	Cbr-mzt-1.1, CBG03284
I:6867179-6869525	I:6858657-6860983	92.55	Cbr-wee-1.1, CBG06567	CBG21906, Cbr-cnc-4, Cbr-cnc-3
I:6858655-6860982	I:6867180-6869526	92.64	CBG21906, Cbr-cnc-4, Cbr-cnc-3	Cbr-wee-1.1, CBG06567
I:7884100-7891151	I:7028193-7035244	99.97	CBG12104, CBG12105, Cbr-viro-2, Cbr-pot-1, CBG26353, CBG05826, Cbr-swt-7	CBG11902, CBG11903, CBG30143, Cbr-paqr-1, CBG06610, Cbr-nhr-178, CBG06612
I:7233822-7241056	I:7245702-7252936	99.96	Cbr-spe-4, Cbr-fut-1, CBG17973, CBG26323, Cbr-adk-1, CBG27367, CBG27601	CBG11945, CBG11946, Cbr-tars-1, CBG06026, Cbr-rap-3
I:7276238-7279267	I:7892152-7895182	100	CBG03133, CBG17962, CBG17136	CBG26592, CBG27617
I:7338216-7343793	I:7344751-7350328	99.6	CBG25559, CBG30033, CBG05994, CBG05993, CBG17119	Cbr-flap-1, CBG06687
I:7523170-7526884	I:7527845-7531559	99.95	CBG12018, Cbr-wip-1, CBG11828	CBG06740
I:8486251-8487568	I:8484615-8485932	99.85	CBG29853	CBG29852
I:8484614-8485932	I:8486251-8487569	99.92	CBG29852	CBG29853
I:8433926-8435390	I:8005537-8007001	99.84	CBG14192	CBG02960
I:8002165-8007001	I:8427483-8432320	99.44	CBG23931, Cbr-pdp-1.1, Cbr-rps-9, CBG02961, CBG02960, CBG05790, CBG08616	CBG12192, Cbr-pdp-1.2, CBG23522
I:8369874-8372703	I:8364987-8367816	99.92	CBG12206, CBG06038	CBG12207
I:8146914-8148994	I:8712277-8714346	87.66	CBG25949	CBG12411
I:8048439-8057284	I:8027971-8036816	99.95	CBG02255, CBG02254, CBG02253, Cbr-bigr-1, Cbr-lir-3, CBG02950, Cbr-str-48, CBG08598, CBG08597	CBG24754, Cbr-hsp-12.1, CBG24756, CBG24757, Cbr-pqm-1, CBG08607, CBG30625, CBG08605
I:8712279-8714362	I:8146901-8148995	87.7	CBG12411	CBG25949
I:9828246-9833872	I:9835223-9840863	99.98	Cbr-nuo-2, CBG30979, Cbr-dhp-2, CBG03445	CBG29118, CBG12673, CBG26395
I:10596874-10605615	I:10606616-10615357	99.96	Cbr-txt-9.1, CBG02319, Cbr-ptr-9, CBG01749, CBG01748, Cbr-set-22, Cbr-snf-10, Cbr-pap-1	CBG02316, CBG31350, CBG02315, Cbr-nhl-1, CBG01746, Cbr-rpl-20, Cbr-cyc-2.1, Cbr-tctn-1, CBG09606
I:11017779-11018528	I:11026776-11027525	99.8	Cbr-msrp-1	Cbr-msrp-6
I:11035842-11039634	I:11078044-11081817	97.83	CBG20251, CBG01632	CBG20242, CBG30532, CBG09452

I:11078047-11081816	I:11035857-11039634	97.81	CBG20242, CBG30532, CBG09452	CBG20251, CBG01632
I:11080655-11081727	I:11231511-11232585	89.94	CBG20242	CBG08500
I:11399713-11403810	I:11571154-11575259	99.12	Cbr-hpo-15	CBG13610, CBG17707, Cbr-str-27, Cbr-odd-2
I:11571173-11575235	I:11399712-11403810	98.95	CBG13610, CBG17707, Cbr-odd-2	Cbr-hpo-15
I:12365185-12369064	I:12507990-12511869	99.99	CBG20401, CBG20402, CBG10926	CBG20432, CBG20433, CBG19627
I:13001070-13005877	I:3457-8335	95.38	CBG19165	Cbr-sqv-8, CBG01066, CBG00325, Cbr-grl-17
I:12722206-12726069	I:12837614-12841445	99.17	Cbr-fipr-24.2, CBG30066, Cbr-map-1	CBG18839
I:12548002-12554217	I:13061341-13067525	99.24	CBG19620, CBG08368, Cbr-odc-1, CBG30805	CBG30069, CBG19495, CBG21194, CBG19186, CBG19187, CBG19188, CBG27815
I:13812723-13815134	I:13809420-13811856	99.59	CBG18489	CBG30593, CBG19371, CBG16882
I:13809433-13811847	I:13812715-13815148	99.88	CBG19371, CBG16882	CBG18489
I:14796277-14799080	I:14801167-14803970	95.19	CBG04652	CBG04655
I:15222383-15223530	I:4963727-4964841	94.57	CBG17036	CBG03918
I:15257988-15265658	I:15249949-15257621	99.79	CBG04781	CBG16021, Cbr-tbx-34, CBG31278, Cbr-mtl-2, CBG04780
II:66677-71747	II:72748-77818	99.86	Cbr-sde-2, CBG11515, CBG08049	CBG00342
II:512064-515307	II:515675-518922	93.72	CBG13813, Cbr-egl-17	CBG26871
II:1216389-1220711	II:1133917-1138239	99.06	CBG30967, CBG00846, CBG15718, CBG16326	CBG00857
II:1121882-1129102	II:1199474-1206680	98.45	CBG18542, Cbr-sfxn-1.2, CBG30324, Cbr-oac-29, CBG16345	CBG30969, Cbr-taco-1, CBG15715
II:1488444-1491181	II:1484260-1486997	99.62	CBG00797	CBG00795
II:1595824-1596950	II:1611602-1612728	99.3	CBG26157	CBG26158
II:1592359-1596028	II:1612521-1616190	99.84	CBG16241	CBG05101, CBG00839, CBG00840, CBG27433
II:1778662-1781943	II:1855128-1858523	99.76	CBG18444, CBG29999, Cbr-josd-1, CBG00487	CBG31002
II:1989928-1991960	II:1994663-1996695	95.07	CBG29812	CBG08875
II:2314636-2315656	II:13733152-13734152	94.26	CBG31124	CBG19348
II:2426866-2430072	II:2434507-2437713	97.35	CBG30439, CBG26638	CBG30496
II:2865336-2870299	II:2901580-2906538	100	CBG25484	CBG22230, CBG13419, CBG13418, CBG22362,

				Cbr-srj-7, Cbr-srj-11, CBG15947
II:2880915-2884271	II:3069972-3073328	99.94	CBG13424, CBG22357, Cbr-srj-9	CBG22271, CBG13380, CBG13379, CBG16983
II:2885288-2892119	II:3262407-3269238	99.63	CBG13423, CBG25829, CBG22359, CBG22421, Cbr-sdz-34.4	CBG15100, CBG13343, Cbr-nmt-1, CBG10514, CBG01894
II:4158933-4168350	II:4314672-4324083	99.64	CBG04167, CBG00542, CBG00543, CBG09040, CBG09039, Cbr-lipl-5, CBG26990, CBG26989, Cbr-nlp-34.4, CBG26988	Cbr-cep-1, Cbr-rpl-25.2, CBG04079, Cbr-wrm-1, CBG30196, CBG26982, CBG26981, CBG02102, CBG02103
II:4144222-4148435	II:4139998-4144211	98.55	CBG00540	CBG04172, Cbr-dnc-6, CBG31324, CBG00538, Cbr-erd-2.2
II:4823536-4828914	II:4834010-4839388	99.63	Cbr-coa-4, CBG01159	CBG04252, CBG31007, CBG31179, CBG21694, Cbr-srj-33, CBG30816
II:4838194-4839388	II:4839399-4840597	98.25	CBG21694	CBG04254
II:4842262-4845929	II:4830114-4833782	99.89	CBG01152, Cbr-ftt-2	CBG01158
II:5304613-5308357	II:5308458-5312191	99.97	CBG12122, CBG12123	CBG11131, Cbr-hex-3, CBG25998, CBG12124
II:5819690-5824660	II:5825661-5830631	99.98	Cbr-stl-1, CBG13080, CBG13183, CBG17917, CBG27385	CBG17919, CBG17920, Cbr-col-177
II:6564597-6570699	II:6576189-6582283	99.98	CBG10822, CBG10823, CBG10824, Cbr-cki-2, CBG18137, CBG30316, CBG27086, Cbr-str-46, CBG06463	Cbr-mat-1, CBG12945, Cbr-pqn-96, Cbr-ufbp-1, Cbr-ifc-1, Cbr-str-39
II:6572050-6576178	II:6582297-6586425	99.99	CBG12946, Cbr-srw-4, Cbr-srw-3	CBG10829, Cbr-pfkb-1.1, Cbr-rpn-3, CBG04479
II:7392256-7393219	II:9662010-9663024	91.8	CBG30348	CBG30738
II:7470239-7475817	II:7484413-7489991	99.64	CBG25565, Cbr-clec-61, CBG05950, CBG05949, Cbr-cpz-2, CBG11812, CBG26804	Cbr-unc-108, CBG03093, CBG03092, CBG25922, Cbr-nhr-8, CBG30726
II:9662013-9663056	II:7392255-7393260	91.6	CBG30738	CBG30348
II:10052028-10056936	II:10057956-10062864	99.96	Cbr-mdt-26, Cbr-ift-43.1, Cbr-rpl-25.1	CBG12716, CBG02468, Cbr-ift-43.2, Cbr-scl-1, Cbr-scl-9
II:10109241-10116736	II:10119713-10127208	99.95	CBG29868, CBG12731, Cbr-cdc-14.2, Cbr-jmjd-3.2, Cbr-hum-10	CBG12733, CBG12734, Cbr-ttc-4, Cbr-cdc-14.1, CBG25640, CBG24368, CBG24369, CBG24371, CBG09743, CBG30526, CBG27068

II:10185727-10189076	II:10192435-10195784	99.79	CBG02428, CBG26018, CBG06900	CBG12747, CBG02426, CBG14554
II:10189076-10191758	II:10198301-10200983	99.95	CBG02427, CBG06899	CBG25369, CBG02425, CBG06896
II:10198299-10200981	II:10189076-10191760	100	CBG25369, CBG02425, CBG06896	CBG02427, CBG06899
II:10281451-10283198	II:10161077-10162798	96.56	CBG02400	Cbr-sec-61.G
II:11375377-11381801	II:11385306-11391730	99.72	CBG08467, CBG08466, CBG25403, CBG20787, CBG20788, CBG14803	CBG08462, CBG25402, CBG20790, CBG20791, CBG09383
II:12296859-12303234	II:12303245-12309620	98.92	Cbr-mtx-2, CBG00034, CBG30446, CBG18975, CBG18976, Cbr-asg-2	Cbr-csn-4, Cbr-gon-14, CBG10907, CBG10908
II:11667151-11673571	II:11680357-11686777	97.8	CBG20860, Cbr-mvb-12, CBG09304, CBG05010	CBG26030, CBG20145, Cbr-ric-3, CBG09301, CBG05015
II:11930967-11935132	II:12407061-12411226	99.8	CBG30118	CBG20410, CBG20411, CBG31464, CBG30650
II:12416274-12422208	II:11935143-11941077	99.86	CBG20414, CBG12246, Cbr-grl-8	CBG17611, Cbr-sdz-13, CBG05074, CBG05075
II:12076340-12077955	II:11933037-11934598	97.26	CBG00002	CBG30118
II:12264279-12266701	II:12423299-12425723	93.86	CBG20995	CBG31280
II:12427940-12429153	II:12430973-12432180	87.31	CBG21037	CBG21039
II:12430977-12432176	II:12427936-12429154	88.91	CBG21039	CBG21037
II:12908766-12912742	II:12439803-12443779	99.94	CBG31528	Cbr-ufm-1
II:12444663-12450908	II:12914549-12920794	99.84	Cbr-rpl-35, Cbr-ugt-59, CBG19012, Cbr-bbs-8	CBG29933, CBG31529, CBG19532, CBG30250, CBG21224, CBG26479, CBG26478, CBG11046
II:12453480-12457714	II:12459869-12464047	98.02	CBG20418, CBG19643, Cbr-srh-30, Cbr-srh-28	CBG19641, Cbr-ugt-44, Cbr-spn-4, Cbr-tsp-16
II:12459833-12464050	II:12453480-12457672	96.3	CBG19641, Cbr-ugt-44, Cbr-spn-4, Cbr-tsp-16	CBG20418, CBG19643, Cbr-srh-30, Cbr-srh-28
II:12466977-12473539	II:12931949-12938511	99.71	CBG20421, CBG19638, CBG08387, CBG19020	CBG21221, Cbr-str-84, CBG19146, CBG11049
II:13018194-13023072	II:13011860-13016763	99.84	CBG19507, CBG19171, CBG30543, CBG19173, Cbr-swm-1	CBG18879, CBG19510, CBG26473, Cbr-nhr-30.1, CBG27177
II:13101976-13105215	II:13106002-13109244	96.42	CBG19486, Cbr-sri-40, CBG10726, CBG19199, CBG19200	CBG19483, CBG26165, CBG21186
II:13106002-13109823	II:13101394-13105215	99.61	CBG19483, CBG26165, CBG21186, CBG30547, Cbr-col-174	CBG19486, Cbr-sri-40, CBG10726, CBG30546, CBG19199, CBG19200
II:13184527-13187950	II:13188328-13191751	99.95	CBG21174, CBG31168	Cbr-atp-2

II:13195818-13199676	II:13165668-13169526	99.94	CBG04349	CBG29899
II:13210515-13212263	II:13201048-13202824	97.81	Cbr-nlp-61	CBG04350
II:12099401-12100628	II:14523348-14524575	92.86	CBG20948	CBG21869
II:14523348-14525105	II:14527276-14529035	93.09	CBG21869	CBG24810
II:14526707-14528432	II:15101545-15103253	89.57	CBG24810	CBG17057
II:15101738-15103253	II:14203501-14205016	87.68	CBG17057	CBG10347
II:14203502-14204860	II:14420582-14421965	92.36	CBG10347	CBG10283
II:14420582-14421793	II:12099489-12100654	86.52	CBG10283	CBG20948
II:13733152-13734152	II:2314652-2315656	95.24	CBG19348	CBG31124
II:13973009-13974032	II:13984297-13985291	86.54	Cbr-ins-32.2	Cbr-prx-11, Cbr-ins-32.5
II:14150197-14151510	II:14143404-14144716	98.17	CBG07912	CBG07911
II:14557696-14560000	II:14568356-14570614	94.38	CBG24149	CBG16447, CBG31150, CBG04587
II:14808172-14809274	II:14871003-14872119	97.77	CBG21442	CBG21457
II:15294684-15296640	II:15284353-15286275	95.88	CBG16034	Cbr-flp-33, CBG16029
II:15284353-15286278	II:15294670-15296630	97.96	Cbr-flp-33, CBG16029	CBG16034
II:16600308-16607497	II:16607799-16614988	99.97	CBG06958, Cbr-drl-1, Cbr-srxa-7	Cbr-dhs-6, CBG30393
III:251831-254547	III:259312-262037	99.93	CBG15234	CBG11878, CBG00994
III:332180-336194	III:336342-340356	99.93	CBG11438, CBG11437	Cbr-srx-42
III:528787-534740	III:534751-540704	98.74	Cbr-str-144	Cbr-catp-3
III:958113-962780	III:1627831-1632509	99.96	CBG11267	CBG15793, CBG08765, CBG16229
III:1649736-1656389	III:1144021-1150674	99.92	Cbr-gsto-3.1, CBG15798, Cbr-srt-44.1, CBG13846, CBG16223	Cbr-gsto-3.2
III:1658437-1664270	III:1538017-1543850	99.94	CBG05095, CBG31062, CBG13848, Cbr-mfsd-11	Cbr-psf-3, CBG29807, CBG00814
III:6811048-6814310	III:1755904-1759159	99.72	CBG21890, CBG30952	CBG00479
III:1880950-1883530	III:1885400-1888002	97.36	CBG22029	Cbr-spp-23
III:3406569-3412108	III:3132111-3137650	98.45	CBG11677, CBG27477	CBG17009, Cbr-sup-10
III:3177475-3184954	III:3184965-3192444	99.52	CBG22296, CBG21090, CBG31277, CBG21093, Cbr-str-164, CBG30649, CBG17019	CBG21094, CBG31276, Cbr-str-163, CBG31037, CBG17021, Cbr-lec-9
III:3936476-3944683	III:3822463-3830670	99.96	CBG04123, CBG04124, CBG29824, Cbr-tofu-4, CBG09097, CBG01426, CBG01425	Cbr-itx-1, CBG09118, Cbr-cutl-23, Cbr-cav-1
III:3940131-3944683	III:3945684-3950236	99.74	CBG29824, Cbr-tofu-4, CBG01425	Cbr-mage-1, CBG09096
III:3983939-3985307	III:4118576-4119916	90.88	CBG09091	CBG09051, CBG30336

III:4529767-4531348	III:4496830-4498393	84.98	CBG05212	CBG08958
III:4557040-4562953	III:4517123-4523036	99.91	Cbr-kbp-3, CBG26141, CBG01239	Cbr-ceh-12, Cbr-col-54, Cbr-nlp-62, CBG30197, CBG21752, CBG21751, CBG01253, Cbr-sav-1
III:4629492-4631486	III:4624163-4626157	99.94	CBG05182	CBG01217
III:4792753-4797708	III:4797809-4802764	99.93	CBG04244, CBG23800, CBG29116, CBG01169	Cbr-ept-1, CBG25513, CBG24620, CBG21708, CBG01168
III:5008803-5010747	III:5130826-5132779	99.28	CBG18376	CBG18345
III:5130808-5132791	III:5008794-5010746	99.23	CBG18345	CBG18376
III:5195994-5198512	III:4989036-4991546	100	CBG18325	Cbr-mrpl-12, CBG18379, CBG01113
III:5570162-5575549	III:6044340-6049727	99.29	Cbr-ikb-1, CBG18240	Cbr-edg-1, CBG13233, Cbr-srbc-58
III:5573187-5575346	III:6049734-6051898	97.56	CBG18240	CBG13234
III:6049737-6051898	III:5573185-5575346	99.78	CBG13234	CBG18240
III:5793019-5797080	III:5798081-5802142	99.97	Cbr-npr-31, CBG30598	CBG30025, Cbr-fust-1
III:5883416-5890760	III:5891471-5898815	99.97	Cbr-bro-1, CBG26571, CBG13099, CBG26150, CBG30027, CBG13100, CBG30843, CBG19948, Cbr-srx-22, Cbr-irld-3, CBG17543, Cbr-nlp-19	Cbr-npp-6, CBG13103, CBG26414, CBG06281, CBG06282, CBG17546
III:6351606-6359943	III:6364385-6372722	99.97	Cbr-pgam-5, CBG13314, Cbr-trap-4.1, CBG13317, CBG06415, CBG06416	Cbr-paf-1, CBG29840, CBG13319, Cbr-ccf-1, Cbr-trap-4.2, CBG13322, Cbr-ges-1, CBG06419
III:6503869-6505020	III:6505647-6506829	95.88	CBG18155, Cbr-rps-25	CBG18154
III:6505649-6506802	III:6503867-6505047	95.88	CBG18154	CBG18155, Cbr-rps-25
III:6809503-6814263	III:1754325-1759159	98.58	CBG18081, CBG30952, Cbr-srv-24	CBG30277, CBG30278, CBG00479
III:7871256-7875249	III:7875575-7879568	99.97	CBG31459, CBG08655	CBG16596, CBG08654, CBG08653, CBG08652
III:9037020-9037720	III:9034754-9035454	99.88	CBG09918	CBG09917
III:9202714-9206259	III:9207273-9210818	99.67	CBG02683, CBG09964, CBG14340	CBG14341
III:9245530-9251347	III:9306132-9311949	99.91	Cbr-slc-25A42, CBG12502, Cbr-lmd-1, Cbr-rsp-5, Cbr-snr-3, Cbr-vps-53, CBG09979, CBG09980, CBG30353, Cbr-ppm-1.H, Cbr-rpl-34	CBG02655, CBG10002, CBG10004, CBG14364, CBG14365
III:9551641-9553240	III:9554139-9555738	99.64	CBG14427	CBG23216

III:9757716-9763607	III:9764252-9770143	99.96	CBG12647, CBG02546, CBG10121	CBG02545, CBG02544, CBG25631, CBG30848, CBG10123, CBG03424
III:10642341-10648145	III:10635536-10641340	99.96	CBG30842, CBG06793, Cbr-aqp-5	CBG12848, CBG02308, CBG06795, Cbr-bus-4, CBG09600, Cbr-vps-41
III:10613619-10620004	III:10606233-10612618	99.84	Cbr-ptp-5.3, CBG02313, Cbr-timm-17B.1, Cbr-hoe-1, Cbr-chw-1, CBG14647, CBG14648	CBG02316, Cbr-nhl-1, CBG01746, Cbr-rpl-20, Cbr-cyc-2.1, CBG30616, CBG09606
III:10573860-10576681	III:9960308-9963129	99.94	CBG02329, CBG27075	CBG29867, CBG23095
III:10157359-10162673	III:10163074-10168388	99.98	CBG25368, CBG06907, CBG24380, CBG09731, CBG27070, Cbr-sec-61.G	CBG02435, CBG06906, CBG24382, CBG24383, CBG09729
III:10234616-10235954	III:10236549-10237922	91.55	Cbr-sdz-1.2	Cbr-sdz-1.1
III:10236550-10237922	III:10234616-10235955	91.55	Cbr-sdz-1.1	Cbr-sdz-1.2
III:10480496-10483611	III:9956160-9959307	97.56	CBG27727, Cbr-dpy-8	Cbr-col-123, CBG23097
III:9950187-9954039	III:10462958-10466810	99.92	CBG23099	CBG12811, Cbr-pho-1, CBG14615
III:9956159-9959307	III:10480499-10483647	99.22	Cbr-col-123, CBG23097	CBG27727, Cbr-dpy-8
III:10864685-10868502	III:10868977-10872770	99.89	CBG20614	CBG20616, CBG03559, CBG27741
III:11685663-11689846	III:11689947-11694129	99.67	CBG20144, CBG17682, CBG05016	CBG31295, CBG09120, CBG27755
III:11758538-11763590	III:11765319-11770361	99.48	CBG13645, CBG30986	CBG17655, CBG17654, Cbr-cest-16
III:11908810-11913422	III:11916636-11921248	99.96	Cbr-rbm-3.1, Cbr-sdz-15, Cbr-nas-33	CBG18681, CBG18680, Cbr-nos-1
III:12529827-12540959	III:12542096-12553228	99.41	CBG19622, Cbr-tyr-1, CBG27775	CBG19621, CBG19620, CBG12272, CBG08369, CBG30957, CBG08368, CBG19039, Cbr-odc-1, CBG27777, CBG30805
III:12676844-12686619	III:12687621-12697405	99.94	CBG19589, Cbr-fut-4, CBG30065, Cbr-gsto-1.1, Cbr-egl-21, Cbr-bgnt-1.1, CBG19078, Cbr-nhr-286, Cbr-nhr-135	CBG18810, CBG26173, CBG19583, Cbr-gsto-1.2, Cbr-otpl-1, CBG11000
III:13666809-13676171	III:13685043-13694405	99.72	CBG15379, CBG26207, Cbr-srx-97, CBG23012, Cbr-cyp-29A4, CBG19333	CBG15382, CBG21425, Cbr-pitr-4, Cbr-sulp-8
III:14396033-14403741	III:14404742-14412450	99.8	Cbr-ztf-30.2, CBG04555, CBG04556, Cbr-srh-17	Cbr-impt-1, CBG08010, CBG10287, Cbr-ztf-30.1, Cbr-gasr-8

III:14513523-14521015	III:14246586-14254078	99.73	CBG08031, CBG26568, Cbr-acbp-4.2, CBG15352, CBG15353	CBG10336, CBG00606, CBG00607, Cbr-acbp-4.1, CBG00609, CBG30888, CBG11604, CBG00082
IV:1-4892	IV:1491435-1496326	99.92	Cbr-srv-16.2	CBG05119, Cbr-srv-16.1, CBG27424
IV:578437-584361	IV:584485-590409	99.65	CBG15159, CBG08170	CBG00936
IV:1302973-1306512	IV:11674712-11678275	97.68	Cbr-mesp-1.4, CBG13754	CBG13626, Cbr-maea-1, Cbr-mesp-1.2, Cbr-zgpa-1, CBG05012
IV:1486287-1490434	IV:1589258-1593405	99.97	CBG18719, CBG13798, CBG00797	CBG27430
IV:13351979-13353578	IV:1612007-1613606	99.85	CBG25419	CBG31306, CBG27433
IV:2135188-2138867	IV:4022649-4026444	87.58	CBG21960, CBG21587, Cbr-lbp-4	CBG04213, Cbr-atg-10, Cbr-ceh-49.1, CBG01404
IV:2135429-2136848	IV:2144091-2145584	88.54	CBG21587	Cbr-ceh-49.3
IV:2141648-2144090	IV:4019497-4021943	97.87	CBG21590	CBG30428, CBG01406
IV:2144086-2145798	IV:4016992-4018701	92.51	Cbr-ceh-49.3	Cbr-ceh-49.6
IV:2147784-2150512	IV:4014234-4016992	93.1	CBG30153, CBG21592, CBG20577, CBG16117	CBG15046, CBG01407
IV:2149919-2151085	IV:2135607-2136753	85.39	Cbr-ceh-49.5	CBG21587
IV:2342084-2348827	IV:2334602-2341345	99.99	CBG30494, CBG30495	CBG25182, CBG11716, Cbr-str-33, CBG24588
IV:2906617-2913515	IV:2914002-2920900	99.65	CBG30002, Cbr-srj-32, CBG22430, CBG22431, Cbr-acdh-10, Cbr-acdh-7	Cbr-ptps-1, CBG22233, CBG25832, CBG22432, CBG22435, CBG15943
IV:2883763-2891178	IV:2875332-2882747	98.44	CBG13423, CBG25829, CBG26939, CBG22421, Cbr-sdz-34.4	CBG22223, CBG13424, CBG22357, CBG15951
IV:11132948-11134166	IV:6677825-6679035	99.1	CBG09440	CBG17252
IV:3264186-3267906	IV:3259773-3263493	99.59	CBG13343	CBG31484
IV:3440110-3446474	IV:3462423-3468787	99.76	CBG23889, CBG24733, CBG01928, CBG01929, CBG01931	Cbr-vha-10, CBG00725, CBG30502, CBG10453
IV:3485721-3491767	IV:3536409-3542455	99.48	CBG00719, CBG17492, CBG31482, CBG01942	Cbr-srg-1, CBG22665
IV:3884873-3886206	IV:3881240-3882573	98.25	CBG15020	CBG15019
IV:3881240-3882573	IV:3884873-3886206	98.35	CBG15019	CBG15020
IV:4014317-4017015	IV:2147783-2150512	93.1	CBG15046, CBG01407	CBG30153, CBG21592, CBG20577, CBG16117
IV:4019568-4021944	IV:2141648-2144091	97.43	CBG01406	CBG21590
IV:4022711-4026444	IV:2135148-2138868	87.55	CBG04213, Cbr-atg-10, Cbr-ceh-49.1, CBG01404	CBG21960, CBG21587, Cbr-lbp-4

IV:4824676-4831176	IV:4831529-4838029	99.81	Cbr-knl-1, Cbr-coa-4, CBG31532	CBG31007, CBG31179, Cbr-srj-33, CBG27518, CBG30816
IV:5103394-5106501	IV:11462602-11465725	96.99	Cbr-mcm-4	Cbr-mrpl-48, Cbr-crn-2
IV:5443607-5446884	IV:5438440-5441717	99.9	Cbr-gpx-1, Cbr-gnrr-7	Cbr-cox-7C
IV:5562657-5566988	IV:5691075-5695406	99.56	CBG03773, CBG11185	CBG03730, CBG03731, CBG17501
IV:6012193-6017354	IV:6019155-6024316	99.93	Cbr-vacl-14, CBG29836, Cbr-cul-4, CBG29759, Cbr-cec-6, CBG06319	CBG08223, Cbr-cnep-1, Cbr-mrt-2, CBG17572
IV:6150338-6151836	IV:6154331-6155758	92.98	CBG19895	CBG19894
IV:6154334-6155788	IV:6150335-6151810	92.46	CBG19894	CBG19895
IV:6227912-6230057	IV:6421797-6423912	98.69	CBG08267	CBG18179
IV:6421799-6423909	IV:6227920-6230058	99.81	CBG18179	CBG08267
IV:6464113-6467538	IV:6528469-6531894	99.98	CBG30211	CBG10807, CBG12958, Cbr-skr-17, CBG12956, CBG09465
IV:6848043-6853757	IV:6794784-6800449	98.19	Cbr-pfd-6, CBG18064, Cbr-ipla-6.1	Cbr-xpa-1, CBG03266, Cbr-ipla-6.2, CBG06540, CBG06541
IV:7081315-7082588	IV:7085439-7086739	88.99	CBG30873, CBG05402	CBG05403
IV:7085448-7086739	IV:7081315-7082602	89.52	CBG05403	CBG30873, CBG05402
IV:8209935-8211451	IV:4123483-4124790	99.77	CBG14145	CBG09047
IV:8181097-8182682	IV:6408545-6409915	82.41	CBG05739	CBG19838
IV:7838392-7839970	IV:7694083-7695703	92.03	CBG05839, CBG05838	CBG05886, CBG05885
IV:7836556-7839988	IV:7831202-7834634	99.12	Cbr-cyn-4, CBG05840, CBG05839, CBG05838, CBG08666, Cbr-lin-32	CBG05843, CBG05842, CBG27616
IV:7836556-7838394	IV:7695933-7697771	96.92	CBG05840, CBG08666	Cbr-col-39, CBG05884, CBG29117
IV:7694107-7695703	IV:7833038-7834634	99.12	CBG05886, CBG05885	CBG05842
IV:7830908-7833040	IV:7695933-7698228	96.92	CBG05843	Cbr-col-39, CBG05884, CBG05883, CBG29117
IV:7696959-7698219	IV:8460130-8461347	95.9	CBG05884, CBG05883	CBG06060, CBG06061
IV:7696089-7698218	IV:7830760-7832836	98.32	Cbr-col-39, CBG05884, CBG05883, CBG29117	CBG05844, CBG05843
IV:7696089-7697780	IV:7836556-7838191	98.42	Cbr-col-39, CBG05884, CBG29117	CBG05840
IV:7833037-7834645	IV:7838392-7839999	90.86	CBG05842	CBG05839, CBG05838
IV:7592978-7595000	IV:7590592-7592614	93.85	Cbr-srg-25	CBG12042, CBG16538, Cbr-kbp-1
IV:7397568-7401079	IV:8370163-8373674	99.81	CBG11987, CBG29941, CBG11789	CBG12206, Cbr-ttr-43

IV:8373898-8377633	IV:7393399-7397134	99.88	CBG09753, CBG30417	CBG16488, CBG16489, CBG06702
IV:7224967-7228549	IV:8364722-8368304	99.95	CBG06658	CBG12207, CBG02869, Cbr-best-7.2
IV:8439845-8444977	IV:8452396-8457528	99.88	CBG02845, CBG06055	CBG06057, CBG06058, Cbr-clc-3
IV:8444338-8444977	IV:7694060-7694699	94.01	CBG06055	CBG05886
IV:9188433-9190210	IV:9195134-9196911	99.27	CBG06246	CBG09962, CBG06247
IV:10258416-10264727	IV:10119515-10125826	99.95	Cbr-unc-32, CBG22318, CBG22319, CBG09704, CBG14567	CBG12733, CBG12734, Cbr-ttc-4, Cbr-cdc-14.1, CBG24368, CBG24369, CBG09743, CBG30526, CBG27068
IV:10157466-10158712	IV:10159933-10161177	81.26	CBG06907	CBG27070
IV:10159935-10161177	IV:10157460-10158708	81.18	CBG27070	CBG06907
IV:10285118-10289239	IV:10310865-10314986	99.52	CBG01845, Cbr-scl-15, Cbr-irld-6, Cbr-srh-55.2	Cbr-clec-141, CBG06864, CBG06863, CBG01837
IV:10314590-10315887	IV:10312949-10314246	98.2	CBG01836	CBG06863, CBG01837
IV:10520075-10521242	IV:10521845-10523013	95.93	CBG01768	CBG01767
IV:10521846-10523014	IV:10520073-10521241	96.01	CBG01767	CBG01768
IV:10306609-10308049	IV:10729123-10730563	100	CBG25644, CBG01838	CBG01707
IV:11051208-11052589	IV:11054362-11055742	92.41	CBG29880, CBG01630	CBG01629
IV:11054362-11055823	IV:11051208-11052589	92.26	CBG01629	CBG29880, CBG01630
IV:11138532-11139817	IV:11598259-11599460	96.94	CBG03615	CBG20840
IV:11339388-11346596	IV:11347597-11354805	99.99	CBG08476, CBG08475, Cbr-kin-5.1, Cbr-nlp-10, CBG31347, CBG09397, Cbr-inx-3.1, CBG14795, CBG14796	CBG20777, Cbr-dmsr-7, CBG09394, CBG09393, CBG14797, CBG14798
IV:11462602-11465725	IV:5103393-5106502	96.83	Cbr-mrpl-48, Cbr-crn-2	Cbr-mcm-4
IV:11598257-11599461	IV:11138552-11139755	96.94	CBG20840	CBG03615
IV:13738028-13739370	IV:13652242-13653583	97.18	CBG18475	CBG18462
IV:14199703-14203180	IV:6748366-6751865	98.17	CBG10348, Cbr-irld-14, Cbr-str-124	CBG29843

IV:14204214-14205410	IV:14669012-14670261	94.4	CBG03512	CBG16471, Cbr-rpoa-12
IV:14373173-14378476	IV:14378577-14383880	99.87	CBG10294, Cbr-pssy-2, CBG04548	CBG10292, Cbr-oig-3, Cbr-unc-61
IV:15073694-15080663	IV:15065737-15072710	99.93	Cbr-cht-2	Cbr-mrps-18B
IV:15864423-15868944	IV:15869987-15874508	99.31	CBG07153, CBG31454, Cbr-srd-21	CBG13950, CBG04993, CBG15449
IV:16334530-16337091	IV:16447608-16450174	99.69	CBG07048	CBG30392, CBG23981
IV:16632589-16638046	IV:16594964-16600421	98.27	CBG30987, CBG27998	CBG06960, CBG10577, Cbr-vamp-7, CBG24406
IV:17221319-17225744	IV:17212941-17217366	99.73	CBG26539, CBG30908	CBG13515
V:599181-600665	V:623616-625191	80.69	CBG08173	CBG08179
V:999067-1003150	V:1003284-1007367	99.9	CBG13705, CBG16370	Cbr-sdhb-1, CBG30300, CBG13706
V:1250682-1251830	V:1239196-1240370	98.89	CBG01506, CBG01507	CBG26907, CBG01498
V:1248721-1249878	V:1241031-1242217	97.48	CBG01504, Cbr-his-63	CBG01499, CBG01500
V:1241052-1242163	V:1248749-1249880	98.42	CBG01499, CBG01500	CBG01504, Cbr-his-63
V:1239217-1240366	V:1250659-1251832	98.89	CBG26907, CBG01498	CBG01506, CBG01507
V:1615509-1617177	V:6736547-6738217	96.99	CBG27434	CBG06519
V:1793491-1798178	V:1799932-1804619	99.34	Cbr-tufm-1, CBG30329, CBG26240, Cbr-nmat-1	Cbr-rps-18
V:11475297-11476910	V:7473277-7474891	98.39	CBG26451	CBG26804
V:2002577-2006499	V:4429198-4433114	98.24	CBG21557, CBG31291, CBG20545	Cbr-cytb-5.2, Cbr-dhod-1, CBG01282, CBG31338, CBG27508
V:2591538-2598335	V:2647224-2654021	99.9	CBG13477, CBG21823	Cbr-clec-137, CBG13463, CBG21836, CBG21837, CBG15990
V:2726539-2730247	V:2479336-2483044	99.91	CBG26113, Cbr-srbc-59	CBG31243
V:3152977-3157304	V:3148108-3152435	99.6	CBG17014	CBG27473
V:3053644-3057499	V:3049778-3053633	99.88	CBG22265, CBG13383, CBG13382, CBG31198, CBG16979, CBG31269	CBG16978
V:3308773-3310341	V:7286915-7288488	98.6	CBG13335	CBG30215, CBG17135
V:3484959-3486836	V:3490509-3492386	99.73	CBG10446	CBG10445
V:3717153-3720587	V:3551416-3554850	99.99	CBG01988, CBG30710, CBG01989, CBG27487	Cbr-wdr-12, CBG10431, CBG10430
V:3576941-3578198	V:3579150-3580410	95.75	CBG10424	CBG10423
V:3579150-3580409	V:3576941-3578199	95.83	CBG10423	CBG10424
V:4256370-4257436	V:4261518-4262553	84.98	CBG00569	CBG01340

V:4429253-4432344	V:2003348-2006498	95.4	Cbr-cytb-5.2, CBG01282, CBG31338, CBG27508	CBG21557, CBG31291, CBG20545
V:4534984-4539853	V:4543559-4548439	97.59	CBG30453, CBG01247, Cbr-nhr-190.1	CBG21747, CBG01244
V:4710542-4711672	V:4732294-4733424	97.62	CBG01197	CBG01188
V:4727914-4729992	V:4714693-4716780	96.69	CBG01191	CBG01195, CBG01194
V:1591124-1595255	V:7018817-7022939	98.87	CBG15784	Cbr-mab-21, CBG26374
V:4963465-4967087	V:4748484-4752106	99.2	CBG03918, CBG29829, Cbr-hsp-12.3	CBG25242, CBG21719
V:4986537-4988441	V:4991276-4993180	98.53	CBG01114	CBG03913, CBG01112
V:5443652-5448955	V:5493285-5498587	99.27	Cbr-gpx-1, CBG20035, Cbr-gnrr-7	Cbr-nubp-1, CBG30963, CBG18258, Cbr-elo-7, CBG27003
V:5553517-5555138	V:5548397-5550061	96	Cbr-nhr-247.2	CBG03776, Cbr-nhr-247.1
V:5548677-5550060	V:5553517-5554908	98.64	CBG03776, Cbr-nhr-247.1	Cbr-nhr-247.2
V:5712617-5717525	V:5877267-5882132	98.66	CBG25223, Cbr-marc-3, CBG29835, CBG17888, Cbr-lpr-5	CBG26570, CBG13096, CBG13192, Cbr-pepm-1.1, CBG30594, CBG17941
V:5873554-5874720	V:5882233-5883412	99.07	CBG17939	CBG06277
V:6097873-6101801	V:7009895-7013828	99.97	CBG06338	Cbr-erh-2, CBG05425, CBG06602
V:6102339-6106551	V:7013985-7018197	99.88	CBG13251, CBG19910, CBG06339, CBG06340	Cbr-asb-1, Cbr-srbc-36, CBG06605
V:6632625-6634060	V:6630383-6631817	99.79	CBG06483	CBG06482
V:6692003-6699160	V:6699653-6706810	99.65	CBG20490, CBG03287, CBG30480, CBG05511, CBG30902, CBG06506	CBG20487, Cbr-best-23, CBG06508, CBG30903, CBG06509, CBG31518
V:6792053-6797063	V:17754576-17759614	96.77	CBG05481, Cbr-srw-103, CBG06539, CBG17232	CBG17824
V:6798082-6807842	V:17742713-17752578	96.99	Cbr-crml-1, Cbr-dnj-23, Cbr-dyrb-1, CBG03263, Cbr-txt-9.2, Cbr-mnp-1, CBG26378, Cbr-ops-1, CBG06541, CBG06542, Cbr-gur-3	CBG17823, CBG30845, CBG30844, Cbr-srw-101.1
V:6805543-6808207	V:17742351-17745029	96.69	Cbr-ops-1	CBG17823
V:6828374-6830854	V:17738590-17741140	95.12	CBG06553	CBG17821
V:7000583-7008876	V:7024096-7032389	99.86	Cbr-col-61, CBG18035, CBG30448, CBG31384, Cbr-ddi-1, CBG05426, CBG06598, CBG06599, CBG06600, CBG06601	CBG21944, CBG11902, CBG11903, Cbr-fan-1, Cbr-paqr-1, CBG06608, CBG06609, CBG06610, Cbr-nhr-178
V:7069686-7079545	V:7503125-7512985	99.69	CBG03194, Cbr-odr-8, CBG05409, Cbr-dnj-11,	Cbr-glh-4, CBG25567, CBG03087, CBG03086, CBG16518, Cbr-tofu-7

			Cbr-lec-11, CBG05406, CBG17182	
V:7286918-7288488	V:3308773-3310341	98.54	CBG30215, CBG17135	CBG13335
V:7473275-7474892	V:5830803-5832411	99.38	CBG26804	CBG25854
V:7496549-7497800	V:12647783-12649040	99.44	Cbr-tmem-17	CBG10990
V:7796605-7800344	V:7808914-7812661	99.92	CBG12082, Cbr-srab-3.2, CBG14047	CBG12086, CBG25575, CBG25932, Cbr-srab-3.1
V:7802613-7808915	V:7812923-7819227	99.92	Cbr-aars-2, CBG31425, Cbr-crn-4	CBG12088, Cbr-mrps-6, CBG03007, CBG05848, CBG08671
V:7839233-7840526	V:3635518-3636828	99.01	CBG05838	Cbr-col-176
V:8035783-8039182	V:8323384-8326783	99.97	CBG25104, CBG25105, Cbr-mutd-1, CBG08604, CBG31468	Cbr-wdr-5.1, CBG24764, CBG24765
V:8688628-8690761	V:8696394-8698533	98.98	CBG23445, CBG23444	Cbr-col-86, CBG23437, CBG23436
V:8690871-8692346	V:8694903-8696394	97.39	CBG23443, CBG23442	CBG23438
V:8694917-8696395	V:8690860-8692345	98.26	CBG23438	CBG23443, CBG23442
V:8696407-8698533	V:8688617-8690762	98.79	Cbr-col-86, CBG23437, CBG23436	CBG23445, CBG23444
V:9396376-9399738	V:9402167-9405544	99.97	Cbr-unc-120	CBG10027
V:9399708-9402130	V:9409565-9411987	99.93	Cbr-mpst-1.2	CBG29920, Cbr-set-4, CBG03338, CBG03339, Cbr-mpst-1.1
V:9549597-9550606	V:9646412-9647413	99.6	Cbr-col-157.2	Cbr-col-157.1
V:9760395-9763760	V:8340151-8343516	94.88	CBG02546	CBG12210, CBG02877
V:8340162-8342434	V:8343618-8345890	95.12	CBG02877	CBG02875
V:8353245-8358744	V:9763771-9769270	99.96	CBG05701, CBG27022, CBG31220	CBG02545, CBG02544, CBG30848, CBG10123, CBG03424, CBG30653
V:10121964-10125612	V:10159335-10162983	99.87	CBG09743, CBG30526, CBG27068	CBG24380, Cbr-dod-24, CBG27070, Cbr-sec-61.G
V:10265605-10266901	V:14102969-14104290	93.06	CBG09703	CBG11554
V:11052533-11060615	V:11060716-11068798	99.98	CBG20245, CBG20667, Cbr-gst-12, CBG01629, CBG01628, CBG01627, CBG29115	Cbr-gst-15, Cbr-gst-24, Cbr-gst-20, Cbr-csc-1, Cbr-fbxc-23, CBG03602, CBG01626, CBG01624, CBG26446
V:11134458-11140118	V:11128430-11134089	99.95	CBG03615, CBG14754	CBG08517, Cbr-max-1.2, CBG09440
V:12476774-12480759	V:12482518-12486503	99.52	CBG20423, CBG19636, CBG12261, CBG19023	CBG30245, CBG26494, Cbr-dmsr-11, Cbr-cah-5
V:12600724-12604044	V:13133560-13136877	99.91	CBG26489	CBG04336

V:12604039-12614128	V:13136872-13146956	99.76	CBG18795, Cbr-nhr-88, CBG12283, CBG26488, CBG30366, CBG30367, CBG19050, CBG19052, CBG19053, Cbr-alh-10	CBG15859, CBG15858, CBG15857, CBG04338, CBG25522, Cbr-gop-2, Cbr-col-103, CBG26472, CBG19209, Cbr-srx-16.2, Cbr-srx-16.1, Cbr-pcyt-1, CBG16734
V:12792386-12798751	V:12803727-12810092	99.78	Cbr-set-11, CBG19561, CBG27169, Cbr-hpo-9, Cbr-hyls-1.1, Cbr-spp-2, Cbr-spp-5, CBG27790, CBG27791	CBG26597, CBG19559, CBG19558, CBG19557, CBG21241, CBG19115, CBG19116, Cbr-hyls-1.2
V:13435507-13438945	V:14044466-14047904	99.13	CBG19274	CBG19434
V:14075721-14077736	V:14533141-14535156	96.95	CBG30297	CBG24814
V:14284794-14286909	V:14364904-14366860	86.38	CBG11614	CBG10301, CBG04543
V:14364989-14366831	V:14284809-14286878	85.28	CBG10301, CBG04543	CBG11614
V:14581636-14583534	V:14635555-14637499	93.79	CBG04591	CBG04607, CBG04608
V:14635538-14637610	V:14581641-14583613	94.8	CBG04607, CBG04608	CBG04591, CBG04592
V:15337441-15338195	V:15335630-15336384	98.76	CBG04806	CBG04804
V:14928785-14929720	V:14904066-14905001	95.86	CBG04710	CBG04696
V:14905700-14908099	V:14902697-14905095	97.33	CBG04698, CBG00220	CBG04695, CBG04696
V:14902678-14905095	V:14905698-14908098	96.42	CBG04695, CBG04696	CBG04698, CBG00220
V:15595271-15598316	V:15521456-15524559	98.11	CBG04880	CBG04857, CBG31263
V:15599123-15600191	V:15644224-15645317	95.26	CBG04882, CBG30796	CBG04902
V:15644224-15645318	V:15519513-15520607	95.75	CBG04902	CBG04855
V:15511106-15517027	V:15602224-15608145	97.43	CBG23572, CBG04852, CBG04853, CBG15530	CBG30089, CBG04884, CBG04885
V:15512562-15515972	V:15640702-15644116	97	CBG04852, CBG04853, CBG15530	CBG04904
V:15695416-15698154	V:15704415-15707157	96.03	CBG30090, CBG04923, CBG27260	CBG04926, CBG31266, CBG27968
V:15782678-15784028	V:15784308-15785658	97.2	CBG04955	CBG04956
V:15784333-15785704	V:15782674-15784028	97.3	CBG04956	CBG04955
V:16560669-16561924	V:16567634-16568855	81.47	CBG16823, CBG24668	CBG24669
V:16577531-16582375	V:16571697-16576541	99.13	Cbr-nstp-4, CBG22558, CBG24401	CBG24357, CBG22733
V:16662858-16669610	V:16746600-16753359	99.87	CBG30395, CBG22717	CBG24530, CBG22701
V:16754883-16756647	V:16761860-16763588	94.75	CBG22915	CBG22913
V:16761860-16763584	V:16754923-16756649	94.81	CBG22913	CBG22915
V:16852460-16853928	V:16885006-16886475	97.89	CBG22885	CBG22876
V:16873731-16877972	V:16920531-16924772	98.54	CBG22880, CBG22879	CBG22500, CBG22501, CBG22867, CBG05232
V:16924286-16927858	V:16877021-16880659	99.31	CBG22865, CBG05233	CBG22878, CBG22877

V:16925393-16929958	V:16930960-16935525	99.16	CBG22865, CBG22864, CBG22863	CBG26532, CBG22862, CBG22861, CBG22860, CBG05235
V:16877021-16879486	V:16930960-16933425	99.16	CBG22878	CBG22862
V:16971167-16972718	V:16972967-16974518	96	CBG22843	CBG22842
V:16998066-17001663	V:17016446-17020043	97.96	CBG30398, CBG31228, CBG31227	CBG22516, CBG22832
V:17194835-17197015	V:17217104-17219283	98.44	CBG18656, CBG28021	CBG18650
V:17197171-17200946	V:17219379-17223154	99.17	CBG13511	CBG26539, CBG18649
V:17252822-17257888	V:17257889-17262955	99.92	Cbr-ham-1, Cbr-str-96.1, CBG28028, CBG05310	CBG18638, Cbr-str-96.2, CBG28029
V:17409522-17411096	V:11475297-11476915	98.64	Cbr-ced-2	CBG26451
V:17738592-17741135	V:6828360-6830856	95.28	CBG17821	CBG06553
V:17742353-17746622	V:6803938-6808211	97.56	CBG17823, CBG30845	Cbr-clh-5, Cbr-ops-1
V:6798057-6803886	V:17746736-17752565	99.21	Cbr-dnj-23, Cbr-dyrb-1, Cbr-txt-9.2, CBG26378, CBG06541, Cbr-gur-3	CBG30844, Cbr-srw-101.1
V:17754557-17759631	V:6792052-6797090	96.75	CBG17824	CBG05481, Cbr-srw-103, CBG06539, CBG17232
V:18073829-18080269	V:18081971-18088411	99.88	CBG23817, CBG23818, CBG07673	CBG27335, CBG23820, CBG07671
V:19227783-19232321	V:19232520-19237058	99.92	Cbr-snx-6	CBG31387, CBG28079, Cbr-cdk-4
V:19348264-19355264	V:19356265-19363265	99.91	Cbr-str-93	CBG05675, Cbr-nlp-53, CBG07404
X:655838-664603	X:591827-600592	99.64	CBG11342, CBG26883, Cbr-ife-2.2	Cbr-dpl-1, CBG13652, CBG11356, CBG31495, Cbr-ife-2.1, CBG30827, CBG08173
X:470451-474130	X:1436403-1440055	98.03	CBG15181, CBG11389	Cbr-cutl-20, CBG26913, CBG00777, CBG16276
X:1436403-1440049	X:6856935-6860571	98.44	Cbr-cutl-20, CBG26913, CBG00777, CBG16276	CBG25467, CBG21906, Cbr-nex-3, Cbr-cnc-11, Cbr-cnc-4
X:160068-161678	X:5494157-5495765	95.77	Cbr-arrd-4	CBG30963
X:1111674-1119550	X:927943-935819	99	CBG29802, CBG29803, CBG29804, Cbr-cus-2, Cbr-nlp-63, CBG01464, CBG31299, Cbr-acd-4, CBG16347	Cbr-strm-1, CBG13699, Cbr-gpa-2
X:1436127-1440008	X:6856933-6860845	98.12	Cbr-cutl-20, CBG00775, CBG26913, CBG16276	CBG25467, CBG21906, Cbr-nex-3, Cbr-cnc-11, Cbr-cnc-4, Cbr-cnc-3
X:1436403-1440013	X:470479-474129	97.96	Cbr-cutl-20, CBG26913, CBG16276	CBG15181, CBG11389

X:1766561-1771904	X:1771915-1777258	99.03	CBG22016	CBG18442
X:2370428-2376403	X:2376575-2382550	99.92	Cbr-elc-1, CBG11706, CBG23597, CBG23599, CBG16068	CBG23602, CBG23603, CBG23605, CBG16067
X:2437112-2444649	X:2445650-2453187	99.78	Cbr-bpnt-1, Cbr-pstk-1, CBG18739, CBG26260, CBG31211	CBG31214, CBG31215, CBG31216, CBG16054
X:3047844-3049586	X:3045681-3047423	91.69	CBG16977	CBG22262
X:3443033-3445309	X:3445816-3448095	99.87	CBG01929, CBG01931	CBG14897, CBG01932, CBG01933
X:3445814-3448089	X:3443036-3445316	99.78	CBG14897, CBG01932, CBG01933	CBG01929, CBG01931
X:3714048-3716038	X:3721080-3723070	99.55	CBG30709	CBG19690
X:3721082-3723080	X:3714049-3716038	99.95	CBG19690	CBG30709
X:4595454-4598017	X:4590125-4592679	96.88	Cbr-elof-1, CBG02162	CBG11101, CBG02161
X:4740312-4741373	X:4738999-4740060	97.87	CBG26290	CBG01185
X:4899445-4905960	X:4906961-4913476	98.92	CBG30895	CBG03932, CBG01134
X:5122661-5126341	X:5127342-5131022	99.49	Cbr-tbca-1, Cbr-hpr-9	CBG27527
X:5494141-5495683	X:160065-161678	95.4	CBG30963	Cbr-arrd-4
X:5697376-5702158	X:5664184-5668965	98.62	Cbr-snpn-1, CBG30837, CBG17503	CBG26416, CBG17881, CBG17448, CBG31519
X:6289151-6292122	X:6293261-6296232	99.63	CBG04526	CBG04525
X:6618586-6620661	X:6698713-6700787	99.38	CBG26631, CBG18125, CBG31402	CBG29924, CBG06508
X:6618059-6620664	X:6698713-6701318	99.61	CBG26631, CBG18125, CBG31402, CBG17262	CBG29924, CBG20487, CBG06508
X:6856874-6860876	X:1436127-1440050	98.09	CBG25467, CBG21906, Cbr-nex-3, Cbr-cnc-5.1, Cbr-cnc-11, Cbr-cnc-4, Cbr-cnc-3	Cbr-cutl-20, CBG00775, CBG26913, CBG00777, CBG16276
X:7259860-7262600	X:7265937-7268677	97.58	Cbr-clec-156.2, CBG17143, CBG17142	CBG11950, CBG17140, CBG17139
X:7256685-7261799	X:7262714-7267881	98.88	CBG31491, Cbr-clec-156.1, Cbr-clec-156.2, CBG17143, CBG17142	CBG11949, CBG03138, CBG17140, CBG17139
X:6681205-6684273	X:7288073-7291259	99.28	CBG31260	CBG17956, CBG17135
X:7373724-7376602	X:7284195-7287072	99.13	Cbr-abf-1	Cbr-spo-11
X:7515512-7517699	X:7440018-7442180	96.44	CBG26807	CBG03104, CBG11803
X:7514141-7517487	X:7497578-7500925	99.88	CBG03083, CBG26807	Cbr-lin-37, Cbr-flp-13, CBG11821, CBG11822
X:7440202-7442180	X:7498947-7500925	99.88	CBG03104, CBG11803	CBG11822

X:7449732-7454768	X:7443695-7448731	99.72	Cbr-pmlr-1, Cbr-clec-143.2, CBG05958, Cbr-cutl-1, CBG11808	CBG29846, CBG11998, CBG29847, CBG03103, CBG06720, CBG11805
X:7498950-7500925	X:7515510-7517485	97.4	CBG11822	CBG26807
X:7503651-7506695	X:7388016-7391057	99.87	CBG03087	CBG05975
X:7619401-7620961	X:7625688-7627251	93.87	CBG27610	CBG03052
X:7625690-7627251	X:7619401-7620960	94.12	CBG03052	CBG27610
X:7748515-7752456	X:9550084-9554025	99.75	Cbr-npp-19, CBG14035, CBG14036	CBG30838, Cbr-rbmx-2, Cbr-cpb-2, CBG10073, CBG27058, CBG23217, CBG14426, CBG14427
X:9646665-9655132	X:7765137-7773604	99.96	Cbr-cuti-1, CBG12616, Cbr-rbbp-5, CBG10096, CBG10097, CBG03396, CBG23184, CBG14454	CBG31426, Cbr-srh-130, CBG27004, Cbr-srh-129, CBG14040
X:8271266-8276985	X:8282749-8288468	99.95	CBG12234, CBG02903, CBG31023	CBG12230, CBG25291, CBG02900, CBG26367, Cbr-egg-1
X:8365343-8367845	X:8356871-8359369	99.2	CBG12207	CBG31220
X:8483578-8487333	X:8685095-8688858	96.13	CBG29852, CBG29853, Cbr-gspd-1	Cbr-srh-16
X:8685090-8688854	X:8483561-8487320	96.49	Cbr-srh-16	CBG29852, CBG29853, Cbr-gspd-1
X:9369751-9372074	X:9656133-9658448	100	CBG12535	Cbr-nlp-48, CBG23182
X:9487031-9490039	X:9491391-9494392	99.87	CBG25907, Cbr-vrp-1, CBG27678	Cbr-blos-4, CBG10053, CBG14412
X:9491382-9494391	X:9487041-9490040	99.87	Cbr-blos-4, CBG10053, CBG14412	CBG25907, Cbr-vrp-1, CBG27678
X:9856224-9863089	X:9867880-9874745	99.96	CBG02525, CBG14491, Cbr-amt-4.1	CBG02522, Cbr-dhs-12, Cbr-gst-2, Cbr-gst-4, CBG03453, CBG14493
X:10156562-10160143	X:10588151-10591732	99.89	CBG25368, CBG06907, CBG24379	CBG30140, Cbr-srd-10
X:10590363-10594065	X:10603200-10606902	99.6	CBG25653	Cbr-irld-34, CBG01748
X:10585966-10587150	X:10615451-10616635	99.94	CBG02324, CBG14645	CBG14647
X:10851780-10859904	X:11347480-11355604	99.95	CBG12895, Cbr-nep-12, Cbr-rpl-7A, Cbr-tald-1, Cbr-srsx-30, Cbr-srsx-29, CBG14696, CBG14697	CBG20777, Cbr-rei-1, Cbr-dmsr-7, CBG09394, CBG09393, CBG30687, CBG14797, CBG14798
X:11136277-11137206	X:11140063-11140992	99.68	CBG14754	CBG14755
X:11340033-11346458	X:11416919-11423344	99.88	CBG08476, CBG08475, Cbr-kin-5.1, Cbr-nlp-10, CBG31347, Cbr-inx-3.1, CBG14795	CBG25399, CBG25676, CBG30239, CBG20201, CBG09375, Cbr-cox-16, Cbr-inx-3.2, CBG14812
X:12756679-12761394	X:12761613-12766328	99.84	CBG30851, CBG19101	CBG08316

X:13349711-13353353	X:13353440-13357082	99.86	CBG29972, CBG17758	CBG17755
X:14045022-14047859	X:13547940-13550777	97.46	CBG19434	CBG26193, CBG19306, Cbr-nstp-10
X:15027347-15032984	X:15033985-15039622	99.95	CBG21494, CBG00244	Cbr-str-52, CBG00245
X:15839420-15840722	X:15840923-15842249	91.29	CBG30555, CBG15457	CBG15456
X:15840932-15842249	X:15839411-15840722	91.14	CBG15456	CBG30555, CBG15457
X:16808152-16809399	X:16816156-16817403	99.05	CBG22684	CBG27301, CBG22683
X:17268644-17272126	X:17274419-17277868	99.65	Cbr-manf-1	Cbr-dml-1, CBG18632, CBG05315
X:17274061-17277870	X:17268677-17272484	99.71	Cbr-dml-1, CBG18632, CBG05315	Cbr-manf-1
X:18945464-18947851	X:19079583-19081966	99.71	CBG07494	CBG05615, CBG07468
X:19561527-19563294	X:13028350-13030054	95.98	CBG07374	CBG30545, CBG16706
X:20776916-20778335	X:20568522-20569942	90.13	CBG10263	CBG10217
X:20681624-20683631	X:21078696-21080710	98.66	CBG10247	CBG08890
X:20568523-20570003	X:20776909-20778314	90.76	CBG10217	CBG10263
X:20565780-20569732	X:20557825-20561777	98.13	CBG10216, CBG10217	CBG10213
X:20557825-20561793	X:20565780-20569729	97.7	CBG10213	CBG10216, CBG10217
X:21115273-21117755	X:20882283-20884765	99.76	CBG08881	CBG08942
X:21078697-21080797	X:20681620-20683632	98.61	CBG08890	CBG10247
X:21169837-21171898	X:21537391-21539452	97.67	CBG10674	CBG10579
X:21149080-21156938	X:21189823-21197681	99.95	CBG10681, CBG10680, CBG31485	CBG10666, CBG10665, CBG10664
X:21438766-21440732	X:21444674-21446639	99.75	CBG10602, CBG10601	CBG10598, CBG10597
X:21443123-21444679	X:21440831-21442387	100	CBG10599	CBG10600
X:21445506-21446609	X:21438767-21439898	99.91	CBG10597	CBG10602
